# Diagnosis and treatment of acute appendicitis: 2020 update of the WSES Jerusalem guidelines

**DOI:** 10.1186/s13017-020-00306-3

**Published:** 2020-04-15

**Authors:** Salomone Di Saverio, Mauro Podda, Belinda De Simone, Marco Ceresoli, Goran Augustin, Alice Gori, Marja Boermeester, Massimo Sartelli, Federico Coccolini, Antonio Tarasconi, Nicola de’ Angelis, Dieter G. Weber, Matti Tolonen, Arianna Birindelli, Walter Biffl, Ernest E. Moore, Michael Kelly, Kjetil Soreide, Jeffry Kashuk, Richard Ten Broek, Carlos Augusto Gomes, Michael Sugrue, Richard Justin Davies, Dimitrios Damaskos, Ari Leppäniemi, Andrew Kirkpatrick, Andrew B. Peitzman, Gustavo P. Fraga, Ronald V. Maier, Raul Coimbra, Massimo Chiarugi, Gabriele Sganga, Adolfo Pisanu, Gian Luigi de’ Angelis, Edward Tan, Harry Van Goor, Francesco Pata, Isidoro Di Carlo, Osvaldo Chiara, Andrey Litvin, Fabio C. Campanile, Boris Sakakushev, Gia Tomadze, Zaza Demetrashvili, Rifat Latifi, Fakri Abu-Zidan, Oreste Romeo, Helmut Segovia-Lohse, Gianluca Baiocchi, David Costa, Sandro Rizoli, Zsolt J. Balogh, Cino Bendinelli, Thomas Scalea, Rao Ivatury, George Velmahos, Roland Andersson, Yoram Kluger, Luca Ansaloni, Fausto Catena

**Affiliations:** 1 Cambridge Colorectal Unit, Cambridge University Hospitals NHS Foundation Trust, Addenbrooke’s Hospital, Cambridge Biomedical Campus, Hills Road, Cambridge, CB2 0QQ UK; 2 Department of General Surgery, University of Insubria, University Hospital of Varese, ASST Sette Laghi, Regione Lombardia, Varese, Italy; 3 Department of General and Emergency Surgery, Cagliari University Hospital, Cagliari, Italy; 4 Emergency and Trauma Surgery Department, Maggiore Hospital of Parma, Parma, Italy; 5 Emergency and General Surgery Department, University of Milan-Bicocca, Milan, Italy; 6 Department of Surgery, University Hospital Centre of Zagreb, Zagreb, Croatia; 7 Maggiore Hospital Regional Emergency Surgery and Trauma Center, Bologna Local Health District, Bologna, Italy; 8 Department of Surgery, University of Amsterdam, Amsterdam, The Netherlands; 9 Macerata Hospital, Macerata, Italy; 10 General, Emergency and Trauma Surgery, Pisa University Hospital, Pisa, Italy; 11 Department of Digestive, Hepato-Pancreato-Biliary Surgery and Liver Transplantation, Henri Mondor University Hospital, Paris, France; 12 Trauma and General Surgeon Royal Perth Hospital & The University of Western Australia, Perth, Australia; 13 Department of Abdominal Surgery, Abdominal Center, University of Helsinki and Helsinki University Central Hospital, Helsinki, Finland; 14 Department of General Surgery, Azienda Socio Sanitaria Territoriale, di Valle Camonica, Italy; 15 Queen’s Medical Center, University of Hawaii, Honolulu, HI USA; 16 Denver Health System – Denver Health Medical Center, Denver, USA; 17 Acute Surgical Unit, Canberra Hospital, ACT, Canberra, Australia; 18 Department of Gastrointestinal Surgery, Stavanger University Hospital, Stavanger, Norway; 19 Department of Surgery, University of Jerusalem, Jerusalem, Israel; 20 Department of Surgery, Radboud University Medical Center, Nijmegen, The Netherlands; 21 Department of Surgery Hospital Universitario, Universidade General de Juiz de Fora, Juiz de Fora, Brazil; 22 Letterkenny Hospital, Donegal, Ireland; 23 Department of Upper GI Surgery, Royal Infirmary of Edinburgh, Edinburgh, Scotland, UK; 24 General, Acute Care, Abdominal Wall Reconstruction, and Trauma Surgery, Foothills Medical Centre, Calgary, Alberta Canada; 25 Department of Surgery, University of Pittsburgh School of Medicine, UPMC-Presbyterian, Pittsburgh, USA; 26 Faculdade de Ciências Médicas (FCM) – Unicamp, Campinas, SP Brazil; 27 Department of Surgery, University of Washington, Harborview Medical Center, Seattle, WA USA; 28 UCSD Health System - Hillcrest Campus Department of Surgery Chief Division of Trauma, Surgical Critical Care, Burns, and Acute Care Surgery, San Diego, CA USA; 29 Department of Emergency Surgery, “A. Gemelli Hospital”, Catholic University of Rome, Rome, Italy; 30 Gastroenterology and Endoscopy Unit, University Hospital of Parma, University of Parma, Parma, Italy; 31 Department of Surgery, Nicola Giannettasio Hospital, Corigliano-Rossano, and La Sapienza University of Rome, Rome, Italy; 32 Department of Surgical Sciences and Advanced Technologies “GF Ingrassia”, Cannizzaro Hospital, University of Catania, Catania, Italy; 33 Niguarda Hospital Trauma Center, Milan, Italy; 34 Department of Surgery, Immanuel Kant Baltic Federal University, Kaliningrad, Russia; 35 Department of Surgery, San Giovanni Decollato Andosilla Hospital, Viterbo, Italy; 36 General Surgery Department, Medical University, University Hospital St George, Plovdiv, Bulgaria; 37 Department of Surgery, Tbilisi State Medical University, TSMU, Tbilisi, Georgia; 38 Section of Acute Care Surgery, Westchester Medical Center, Department of Surgery, New York Medical College, Valhalla, NY USA; 39 Department of Surgery, College of Medicine and Health Sciences, UAE University, Al-Ain, United Arab Emirates; 40 Bronson Trauma Surgery, Kalamazoo, USA; 41 Hospital de Clinicas, Universidad Nacional de Asuncion, Asuncion, Paraguay; 42 Surgical Clinic, Department of Experimental and Clinical Sciences, University of Brescia, Brescia, Italy; 43 Hospital universitario de Alicante, departamento de Cirugia General, Alicante, Spain; 44 Department of Surgery, St. Michael Hospital, University of Toronto, Toronto, Canada; 45 Department of Traumatology, John Hunter Hospital and University of Newcastle, Newcastle, NSW Australia; 46 R. Adams Cowley Trauma Center, Baltimore, MD USA; 47 Professor Emeritus Virginia Commonwealth University, Richmond, VA USA; 48 Harvard Medical School, Massachusetts General Hospital, Boston, USA; 49 Department of Surgery, Linkoping University, Linkoping, Sweden; 50 Division of General Surgery, Rambam Health Care Campus, Haifa, Israel; 51 Department of General Surgery and Trauma, Bufalini Hospital, Cesena, Italy

**Keywords:** Acute appendicitis, Appendicitis guidelines, Jerusalem guidelines, Consensus conference, Alvarado score, Appendicitis diagnosis score, Adult Appendicitis Score, Imaging, CT scan appendicitis, Non-operative management, Antibiotics, Complicated appendicitis, Appendectomy, Laparoscopic appendectomy, Diagnostic laparoscopy, Phlegmon, Appendiceal abscess

## Abstract

**Background and aims:**

Acute appendicitis (AA) is among the most common causes of acute abdominal pain. Diagnosis of AA is still challenging and some controversies on its management are still present among different settings and practice patterns worldwide.

In July 2015, the World Society of Emergency Surgery (WSES) organized in Jerusalem the first consensus conference on the diagnosis and treatment of AA in adult patients with the intention of producing evidence-based guidelines. An updated consensus conference took place in Nijemegen in June 2019 and the guidelines have now been updated in order to provide evidence-based statements and recommendations in keeping with varying clinical practice: use of clinical scores and imaging in diagnosing AA, indications and timing for surgery, use of non-operative management and antibiotics, laparoscopy and surgical techniques, intra-operative scoring, and peri-operative antibiotic therapy.

**Methods:**

This executive manuscript summarizes the WSES guidelines for the diagnosis and treatment of AA. Literature search has been updated up to 2019 and statements and recommendations have been developed according to the GRADE methodology. The statements were voted, eventually modified, and finally approved by the participants to the consensus conference and by the board of co-authors, using a Delphi methodology for voting whenever there was controversy on a statement or a recommendation. Several tables highlighting the research topics and questions, search syntaxes, and the statements and the WSES evidence-based recommendations are provided. Finally, two different practical clinical algorithms are provided in the form of a flow chart for both adults and pediatric (< 16 years old) patients.

**Conclusions:**

The 2020 WSES guidelines on AA aim to provide updated evidence-based statements and recommendations on each of the following topics: (1) diagnosis, (2) non-operative management for uncomplicated AA, (3) timing of appendectomy and in-hospital delay, (4) surgical treatment, (5) intra-operative grading of AA, (6) ,management of perforated AA with phlegmon or abscess, and (7) peri-operative antibiotic therapy.

## Background

Acute abdominal pain accounts for 7–10% of all emergency department accesses [1]. Acute appendicitis (AA) is among the most common causes of lower abdominal pain leading patients to attend the emergency department and the most common diagnosis made in young patients admitted to the hospital with an acute abdomen.

The incidence of AA has been declining steadily since the late 1940s. In developed countries, AA occurs at a rate of 5.7–50 patients per 100,000 inhabitants per year, with a peak between the ages of 10 and 30 [2, 3].

Geographical differences are reported, with a lifetime risk for AA of 9% in the USA, 8% in Europe, and 2% in Africa [4]. Moreover, there is great variation in the presentation, severity of the disease, radiological workup, and surgical management of patients having AA that is related to country income [5].

The rate of perforation varies from 16% to 40%, with a higher frequency occurring in younger age groups (40–57%) and in patients older than 50 years (55–70%) [6].

Appendiceal perforation is associated with increased morbidity and mortality compared with non-perforating AA. The mortality risk of acute but not gangrenous AA is less than 0.1%, but the risk rises to 0.6% in gangrenous AA. On the other hand, perforated AA carries a higher mortality rate of around 5%. Currently, growing evidence suggests that perforation is not necessarily the inevitable result of appendiceal obstruction, and an increasing amount of evidence now suggests not only that not all patients with AA will progress to perforation, but even that resolution may be a common event [7].

The clinical diagnosis of AA is often challenging and involves a synthesis of clinical, laboratory, and radiological findings. The diagnostic workup could be improved by using clinical scoring systems that involve physical examination findings and inflammatory markers. Many simple and user-friendly scoring systems have been used as a structured algorithm in order to aid in predicting the risk of AA, but none has been widely accepted [8–10]. The role of diagnostic imaging, such as ultrasound (US), computed tomography (CT), or magnetic resonance imaging (MRI), is another major controversy [11, 12].

Since surgeons started performing appendectomies in the nineteenth century, surgery has been the most widely accepted treatment, with more than 300,000 appendectomies performed annually in the USA [13]. Current evidence shows laparoscopic appendectomy (LA) to be the most effective surgical treatment, being associated with a lower incidence of wound infection and post-intervention morbidity, shorter hospital stay, and better quality of life scores when compared to open appendectomy (OA) [14, 15].

Despite all the improvements in the diagnostic process, the crucial decision as to whether to operate or not remains challenging. Over the past 20 years, there has been renewed interest in the non-operative management of uncomplicated AA, probably due to a more reliable analysis of postoperative complications and costs of surgical interventions, which are mostly related to the continuously increasing use of minimally invasive techniques [16–18].

The most common postoperative complications, such as wound infection, intra-abdominal abscess, and ileus, vary in frequency between OA (overall complication rate of 11.1%) and LA (8.7%) [19].

In August 2013, the Organizational Board of the 2nd World Congress of the World Society of Emergency Surgery (WSES) endorsed its president to organize the first Consensus Conference on AA, in order to develop the WSES Guidelines on this topic. The Consensus Conference on AA was held in Jerusalem, Israel, on July 6, 2015, during the 3rd World Congress of the WSES, following which, the WSES Jerusalem guidelines for diagnosis and treatment of AA were published [20].

Over the last 4 years, major issues still open to debate in the management of AA have been reported concerning the timing of appendectomy, the safety of in-hospital delay, and the indications to interval appendectomy following the resolution of AA with antibiotics [21–24]. Therefore, the board of the WSES decided to convene an update of the 2016 Jerusalem guidelines.

## Materials and methods

These updated consensus guidelines were written under the auspices of the WSES by the acute appendicitis working group.

The coordinating researcher (S. Di Saverio) invited six experienced surgeons (G. Augustin, A. Birindelli, B. De Simone, M. Podda, M. Sartelli, and M. Ceresoli) with high-level experience in the management of AA to serve as experts in this 2020 update of the WSES Jerusalem guidelines. The experts reviewed and updated the original list of key questions on the diagnosis and treatment of AA addressed in the previous version of the guidelines. The subject of AA was divided into seven main topics: (1) diagnosis, (2) non-operative management of uncomplicated AA, (3) timing of appendectomy and in-hospital delay, (4) surgical treatment, (5) intra-operative grading of AA, (6) management of perforated AA with phlegmon or abscess, and (7) antibiotic prophylaxis and postoperative antibiotic treatment.

Both adults and pediatric populations were considered and specific statements and recommendations were made for each of two groups. Pediatric patients were defined as including children and adolescents aged between 1 and 16 years old. Infants were excluded from this review.

Based upon the list of topics, research questions (Patients/Population, Intervention/Exposure, Comparison, Outcome (PICO)) were formulated, reviewed, and adopted as guidance to conduct an exploratory literature search (Table 1).

**Table 1:** Research topics and key questions

Topic	Key questions
**1. Diagnosis**	Q.1.1: What is the value of clinical scoring systems in the management of adult patients with suspected appendicitis? Can they be used as a basis for a structured management? Q.1.2: In pediatric patients with suspected acute appendicitis could the diagnosis be based only on clinical scores? Q.1.3: What is the role of serum biomarkers in evaluating adult patients presenting with clinical features evocative of acute appendicitis? Q.1.4: What is the role of serum biomarkers in evaluating pediatric patients presenting clinical features highly suggestive of acute appendicitis? Q.1.5: What is the optimum pathway for imaging in adult patients with suspected acute appendicitis? Q.1.6: What is the optimum pathway for imaging in pediatric patients with suspected acute appendicitis?
**2. Non-operative management of uncomplicated acute appendicitis**	Q.2.1: Is non-operative management with or without antibiotics a safe and effective treatment option for adult patients with uncomplicated acute appendicitis? Q.2.2: Is non-operative management with or without antibiotics a safe and effective treatment option for pediatric patients with uncomplicated acute appendicitis? Q.2.3: What is the best non-operative management of patients with uncomplicated acute appendicitis?
**3. Timing of appendectomy and in-hospital delay**	Q.3.1: Does in-hospital delay increase the rate of complications or perforation for adult patients with uncomplicated acute appendicitis? Q.3.2: Does in-hospital delay increase the rate of complications or perforation for pediatric patients with uncomplicated acute appendicitis?
**4. Surgical treatment**	Q.4.1: Does laparoscopic appendectomy confer superior outcomes compared with open appendectomy for adult patients with acute appendicitis? Q.4.2: Does laparoscopic appendectomy confer superior outcomes compared with open appendectomy for pediatric patients with acute appendicitis? Q.4.3: Does laparoscopic single-incision surgery confer any advantage over the three-trocar technique in performing laparoscopic appendectomy for adult patients with acute appendicitis? Q.4.4: Does laparoscopic single-incision surgery confer any advantage over the three-trocar technique in performing laparoscopic appendectomy for pediatric patients with acute appendicitis? Q.4.5: Is outpatient laparoscopic appendectomy safe and feasible for patients with uncomplicated acute appendicitis? Q.4.6: Is laparoscopic appendectomy indicated over open appendectomy in specific patient groups? Q.4.7: Does aspiration alone confer clinical advantages over lavage and aspiration for patients with complicated acute appendicitis? Q.4.8: Does the type of mesoappendix dissection technique (endoclip, endoloop, electrocoagulation, Harmonic Scalpel, or LigaSure) produce different clinical outcomes for patients with acute appendicitis undergoing appendectomy? Q.4.9: Does the type of stump closure technique (stapler or endoloop, ligation or invagination of the stump) produce different clinical outcomes for patients with acute appendicitis undergoing appendectomy? Q.4.10: Is the use of abdominal drains recommended after appendectomy for complicated acute appendicitis in adult patients? Q.4.11: Is the use of abdominal drains recommended after appendectomy for complicated acute appendicitis in pediatric patients? Q.4.12: What are the best methods to reduce the risk of SSI in open appendectomies with contaminated/dirty wounds?
**5. Intra-operative grading of acute appendicitis**	Q.5.1: What is the value of scoring systems for intra-operative grading of acute appendicitis? Q.5.2: Should the macroscopically normal appendix be removed during laparoscopy for acute right iliac fossa pain when no other explanatory pathology is found?
**6. Management of perforated appendicitis with phlegmon or abscess**	Q.6.1: Is early appendectomy an appropriate treatment compared with delayed appendectomy for patients with perforated acute appendicitis with phlegmon or abscess? Q.6.2: Is interval appendectomy always indicated for patients with acute appendicitis following successful NOM?
**7. Perioperative antibiotic therapy**	Q.7.1: Is preoperative antibiotic therapy recommended for patients with acute appendicitis? Q.7.2: Are postoperative antibiotics always indicated in adult patients following appendectomy? Q.7.3: Are postoperative antibiotics always indicated in pediatric patients following appendectomy?

The searches were conducted in cooperation with a medical information specialist from the University of Bologna (A. Gori). A computerized search of different databases (MEDLINE, Scopus, Embase, Web of Science, and the Cochrane Central Register of Controlled Trials), and new citations were included for the period April 2015 to June 2019. No search restrictions were imposed. Search syntaxes have been reported in (Supplemetary material file 1).

The search results were selected and categorized to allow comprehensive published abstract of randomized clinical trials, non-randomized studies, consensus conferences, congress reports, guidelines, government publications, systematic reviews, and meta-analyses.

In the 2016 Jerusalem guidelines, the Oxford classification was used to grade the evidence level (EL) and the grade of recommendation (GoR) for each statement. In this updated document, quality of evidence and strength of recommendations have been evaluated according to the Grading of Recommendations, Assessment, Development and Evaluation (GRADE) system.

The GRADE system is a hierarchical, evidence-based tool, which systematically evaluates the available literature and focuses on the level of evidence based upon the types of studies included. The quality of evidence (QoE) can be marked as high, moderate, low, or very low. This could be either downgraded in case of significant bias or upgraded when multiple high-quality studies showed consistent results. The highest quality of evidence studies (systematic reviews with meta-analysis of randomized controlled trials) was assessed first. If the meta-analysis was of sufficient quality, it was used to answer the research question. If no meta-analysis of sufficient quality was found, randomized controlled trials (RCTs) and non-randomized cohort studies (n-RCS) were evaluated. The strength of the recommendation (SoR) was based on the level of evidence and qualified as weak or strong (Table 2) [25–28].

**Table 2:** GRADE Quality of evidence and strength of recommendations

Quality of evidence and strength of recommendation	Clarity of balance between desirable and undesirable effects	Methodological quality of supporting evidence	Implications
High-quality evidence, strong recommendation	Desirable effects clearly outweigh undesirable effects or vice versa	Consistent evidence from well-performed RCTs or exceptionally strong evidence from unbiased observational studies	Recommendation can apply to most patients in most circumstances. Further research is unlikely to change our confidence in the estimate effect
Moderate quality evidence, strong recommendation	Desirable effects clearly outweigh undesirable effects or vice versa	Evidence from RCTs with important limitations (inconsistent results, methodological flaws, indirectness, imprecision) or exceptionally strong evidence from unbiased observational studies	Recommendation can apply to most patients in most circumstances. Further research (if performed) is likely to have an important impact on our confidence in the estimate of effect and may change the estimate
Low-quality evidence, strong recommendation	Desirable effects clearly outweigh undesirable effects or vice versa	Evidence for at least one critical outcome from observational studies, RCTs with serious flaws or indirect evidence	Recommendation may change when higher quality evidence becomes available. Further research (if performed) is likely to have an important impact on our confidence in the estimate of effect and is likely to change the estimate
Very low-quality evidence, strong recommendation (rarely applicable)	Desirable effects clearly outweigh undesirable effects or vice versa	Evidence for at least one critical outcome from unsystematic clinical observations or very indirect evidence	Recommendation may change when higher quality evidence becomes available; any estimate of effect for at least one critical outcome is very uncertain
High-quality evidence, weak recommendation	Desirable effects closely balanced with undesirable effects	Consistent evidence from well-performed RCTs or exceptionally strong evidence from unbiased observational studies	The best action may differ depending on circumstances or patients or societal values. Further research is unlikely to change our confidence in the estimate effect
Moderate quality evidence, weak recommendation	Desirable effects closely balanced with undesirable effects	Evidence from RCTs with important limitations (inconsistent results, methodological flaws, indirectness, imprecision) or exceptionally strong evidence from unbiased observational studies	Alternative approaches likely to be better for some patients under some circumstances. Further research (if performed) is likely to have an important impact on our confidence in the estimate of effect and may change the estimate
Low-quality evidence, weak recommendation	Uncertainty in the estimates of desirable effects, harms, and burden; desirable effects, harms, and burden may be closely balanced	Evidence for at least one critical outcome from observational studies, RCTs with serious flaws or indirect evidence	Other alternatives may be equally reasonable. Further research is very likely to have an important impact on our confidence in the estimate of effect and is likely to change the estimate
Very low-quality evidence, weak recommendation	Major uncertainty in the estimates of desirable effects, harms, and burden; desirable effects may or may not be balanced with undesirable effects	Evidence for at least one critical outcome from unsystematic clinical observations or very indirect evidence	Other alternatives may be equally reasonable. Any estimate of effect, for at least one critical outcome, is very uncertain

The first draft of the updated statements and recommendations was commented on by the steering group of the guidelines and the board of governors of the WSES during the 6th WSES congress held in Nijmegen, Holland (26–28 June 2019). Amendments were made based upon the comments, from which a second draft of the consensus document was generated. All finalized statements and recommendations with QoE and SoR were entered into a web survey and distributed to all the authors and the board of governor’s members of the WSES by e-mail. The web survey was open from December 1, 2019, until December 15, 2019. The authors were asked to anonymously vote on each statement and recommendation and indicate if they agreed, (≥ 70% “yes” was categorized as agreement), leading to the final version of the document.

## Results

The literature search yielded 984 articles. The titles, abstracts, and full text were reviewed. In total, 157 articles were selected and reviewed in detail to define 48 statements and 51 recommendations addressing seven topics and 30 research questions. A summary of the updated 2020 guidelines statements and recommendations has been reported in Table 3.

**Table 3:** Summary of the updated 2020 guidelines statements and recommendations

Topic	Statement	Recommendation
**1. Diagnosis**	**Statement 1.1** Establishing the diagnosis of acute appendicitis based on clinical presentation and physical examination may be challenging. As the value of individual clinical variables to determine the likelihood of acute appendicitis in a patient is low, a tailored individualized approach is recommended, depending on disease probability, sex, and age of the patient.	**Recommendation 1.1** We recommend to adopt a tailored individualized diagnostic approach for stratifying the risk and disease probability and planning an appropriate stepwise diagnostic pathway in patients with suspected acute appendicitis, depending on age, sex and clinical signs and symptoms of the patient [QoE: Moderate; Strength of recommendation: Strong; 1B].
	**Statement 1.2** Clinical scores alone, e.g. Alvarado score, AIR score, and the new Adult Appendicitis Score are sufficiently sensitive to exclude acute appendicitis, accurately identifying low-risk patients and decreasing the need for imaging and the negative appendectomy rates in such patients.	**Recommendation 1.2.1** We recommend the use of clinical scores to exclude acute appendicitis and identify intermediate-risk patients needing of imaging diagnostics [QoE: High; Strength of recommendation: Strong; 1A]. **Recommendation 1.2.2** We suggest not making the diagnosis of acute appendicitis in pregnant patients on symptoms and signs only. Laboratory tests and inflammatory serum parameters should always be requested [QoE: Very Low; Strength of recommendation: Weak; 2C].
	**Statement 1.3** The Alvarado score is not sufficiently specific in diagnosing acute appendicitis in adults, seems unreliable in differentiating complicated from uncomplicated acute appendicitis in elderly patients and is less sensitive in patients with HIV.	**Recommendation 1.3** We suggest against the use of Alvarado score to positively confirm the clinical suspicion of acute appendicitis in adults [QoE: Moderate; Strength of recommendation: Weak; 2B].
	**Statement 1.4** The AIR score and the AAS score seem currently to be the best performing clinical prediction scores and have the highest discriminating power in adults with suspected acute appendicitis. The AIR and AAS scores decrease negative appendectomy rates in low-risk groups and reduce the need for imaging studies and hospital admissions in both low and intermediate-risk groups.	**Recommendation 1.4** We recommend the use of AIR score and AAS score as clinical predictors of acute appendicitis [QoE: High; Strength of recommendation: Strong; 1A].
	**Statement 1.5** In pediatric patients with suspected acute appendicitis, the Alvarado score and Pediatric Appendicitis Score are useful tools in excluding acute appendicitis.	**Recommendation 1.5** In pediatric patients with suspected acute appendicitis, we suggest against making a diagnosis based on clinical scores alone [QoE: Low; Strength of recommendation: Weak: 2C].
	**Statement 1.6** Biochemical markers represent a promising reliable diagnostic tool for the identification of both negative cases or complicated acute appendicitis in adults. However, further high-quality evidence is needed [QoE: Low; No recommendation].	
	**Statement 1.7** White blood cell count, the differential with the calculation of the absolute neutrophil count, and the CRP are useful lab tests in predicting acute appendicitis in children; moreover, CRP level on admission ≥ 10 mg/L and leucocytosis ≥ 16,000/mL are strong predictive factors for appendicitis in pediatric patients.	**Recommendation 1.6.1** In evaluating children with suspected appendicitis, we recommend to request routinely laboratory tests and serum inflammatory biomarkers [QoE: Very Low; Strength of recommendation: Strong: 1D]. **Recommendation 1.6.2** In pediatric patients with suspected acute appendicitis, we suggest adopting both biomarker tests and scores in order to predict the severity of the inflammation and the need for imaging investigation [QoE: Very Low; Strength of recommendation: Weak: 2D].
	**Statement 1.8** Combination of US and clinical (e.g. AIR, AAS scores) parameters forming combined clinico-radiological scores may significantly improve diagnostic sensitivity and specificity and eventually replace the need for a CT scan in adult patients with suspected acute appendicitis.	**Recommendation 1.7** We recommend the routine use of a combination of clinical parameters and US to improve diagnostic sensitivity and specificity and reduce the need for CT scan in the diagnosis of acute appendicitis. The use of imaging diagnostics is recommended in patients with suspected appendicitis after an initial assessment and risk stratification using clinical scores [QoE: Moderate; Strength of recommendation: Strong; 1B].
	**Statement 1.9** Intermediate-risk classification identifies patients likely to benefit from observation and systematic diagnostic imaging.	**Recommendation 1.8** We suggest proceeding with timely and systematic diagnostic imaging in patients with intermediate-risk of acute appendicitis [QoE: Moderate; Strength of recommendation: Weak; 2B].
	**Statement 1.10** Patients with strong signs and symptoms and high-risk of appendicitis according to AIR score/Alvarado score/AAS score and younger than 40 years may not require cross-sectional pre-operative imaging (i.e., CT scan).	**Recommendation 1.9** We suggest that cross-sectional imaging (i.e., CT scan) in high-risk patients younger than 40 years old (with AIR score 9–12 and Alvarado score 9–10 and AAS ≥ 16) may be avoided before proceeding to diagnostic +/− therapeutic laparoscopy [QoE: Moderate; Strength of recommendation: Weak; 2B].
	**Statement 1.11** POCUS (Point-of-care Ultrasound) is a reliable initial investigation with satisfactory sensitivity and specificity in diagnosing acute appendicitis, easing swift decision-making by the emergency physicians or surgeons. POCUS, if performed by an experienced operator, should be considered the most appropriate first-line diagnostic tool in both adults and children.	**Recommendation 1.10** We recommend POCUS as the most appropriate first-line diagnostic tool in both adults and children, if an imaging investigation is indicated based on clinical assessment [QoE: Moderate; Strength of recommendation: Strong; 1B].
	**Statement 1.12** When it is indicated, contrast-enhanced low-dose CT scan should be preferred over contrast-enhanced standard-dose CT scan. Diagnostic accuracy of contrast-enhanced low-dose CT is not inferior to standard CT in diagnosing AA or distinguishing between uncomplicated and complicated acute appendicitis and enables significant radiation dose reduction.	**Recommendation 1.11** We recommend the use of contrast-enhanced low-dose CT scan over contrast-enhanced standard-dose CT scan for adolescents and young adults with suspected acute appendicitis and negative US findings [QoE: High; Strength of recommendation: Strong; 1A].
	**Statement 1.13** In patients with normal investigations and symptoms unlikely to be acute appendicitis but which do not settle, cross-sectional imaging is recommended before surgery. Laparoscopy is recommended to establish/exclude the diagnosis of acute appendicitis and eventually treat the disease.	**Recommendation 1.12** We recommend cross-sectional imaging before surgery for patients with normal investigations but non-resolving right iliac fossa pain. After negative imaging, initial non-operative treatment is appropriate. However, in patients with progressive or persistent pain, explorative laparoscopy is recommended to establish/exclude the diagnosis of acute appendicitis or alternative diagnoses [QoE: High; Strength of recommendation: Strong; 1A].
	**Statement 1.14** MRI is sensitive and highly specific for the diagnosis of acute appendicitis during pregnancy. However, a negative or inconclusive MRI does not exclude appendicitis and surgery should be still considered if high clinical suspicion.	**Recommendation 1.13.1** We suggest graded compression trans-abdominal ultrasound as the preferred initial imaging method for suspected acute appendicitis during pregnancy [QoE: Very Low; Strength of Recommendation: Weak; 2C]. **Recommendation 1.13.2** We suggest MRI in pregnant patients with suspected appendicitis, if this resource is available, after inconclusive US [QoE: Moderate; Strength of recommendation: Weak; 2B].
	**Statement 1.15** The use of US in children is accurate and safe in terms of perforation rates, emergency department re-visits, and negative appendectomy rates. CT use may be decreased by using appropriate clinical and/or staged algorithm with US/MRI. MRI has at least the same sensitivity and specificity as CT and, although higher costs, should be preferred over CT as second-line imaging in children.	**Recommendation 1.14.1** In pediatric patients with suspected appendicitis, we suggest the use of US as first-line imaging. In pediatric patients with inconclusive US, we suggest choosing the second-line imaging technique based on local availability and expertise, as there are currently no strong data to suggest a best diagnostic pathway due to a variety of options and dependence on local resources [QoE: Moderate; Strength of recommendation: Weak: 2B]. **Recommendation 1.14.2** Since in pediatric patients with equivocal CT finding the prevalence of true acute appendicitis is not negligible, we suggest against the routine use of CT as first-line imaging in children with right iliac fossa pain [QoE: Moderate; Strength of recommendation: Weak; 2B].
**2. Non-operative management of uncomplicated acute appendicitis.**	**Statement 2.1** The antibiotic-first strategy can be considered safe and effective in selected patients with uncomplicated acute appendicitis. Patients who wish to avoid surgery must be aware of a risk of recurrence of up to 39% after 5 years. Most recent data from meta-analyses of RCTs showed that NOM with antibiotics achieves a significantly lower overall complication rate at 5 years and shorter sick leave compared to surgery.	**Recommendation 2.1.1** We recommend discussing NOM with antibiotics as a safe alternative to surgery in selected patients with uncomplicated acute appendicitis and absence of appendicolith, advising of the possibility of failure and misdiagnosing complicated appendicitis [QoE: High; Strength of Recommendation: Strong; 1A]. **Recommendation 2.1.2** We suggest against treating acute appendicitis non-operatively during pregnancy until further high-level evidence is available [QoE: Very Low; Strength of Recommendation: Weak; 2C].
	**Statement 2.2** NOM for uncomplicated acute appendicitis in children is feasible, safe and effective as initial treatment. However, failure rate increases in the presence of appendicolith, and surgery is recommended in such cases.	**Recommendation 2.2** We suggest discussing NOM with antibiotics as a safe and effective alternative to surgery in children with uncomplicated acute appendicitis in the absence of an appendicolith, advising of the possibility of failure and misdiagnosing complicated appendicitis [QoE: Moderate; Strength of recommendation: Weak; 2B].
	**Statement 2.3** Current evidence supports initial intravenous antibiotics with subsequent conversion to oral antibiotics until further evidence from ongoing RCT is available.	**Recommendation 2.3** In the case of NOM, we recommend initial intravenous antibiotics with a subsequent switch to oral antibiotics based on patient's clinical conditions [QoE: Moderate; Strength of recommendation: Strong; 1B].
	**Statement 2.4** Uncomplicated acute appendicitis may safely resolve spontaneously with similar treatment failure rates, shorter length of stay and costs compared with antibiotics. However, there is still limited data for the panel to express in favor of or against the symptomatic treatment without antibiotics [QoE: Moderate; No recommendation].	
**3. Timing of appendectomy and in-hospital delay**	**Statement 3.1** Short, in-hospital surgical delay up to 24 h is safe in uncomplicated acute appendicitis and does not increase complications and/or perforation rate in adults. Surgery for uncomplicated acute appendicitis can be planned for the next available list minimizing delay wherever possible (better patient comfort, etc.). Short, in-hospital delay with observation and repeated trans-abdominal US in pregnant patients with equivocal appendicitis is acceptable and does not seem to increase the risk of maternal and fetal adverse outcomes.	**Recommendation 3.1** We recommend planning laparoscopic appendectomy for the next available operating list within 24 h in case of uncomplicated acute appendicitis, minimizing the delay wherever possible [QoE: Moderate; Strength of recommendation: Strong; 1B].
	**Statement 3.2** Delaying appendectomy for uncomplicated acute appendicitis for up to 24 h after admission does not appear to be a risk factor for complicated appendicitis, postoperative surgical site infection or morbidity. Conversely, appendectomies performed after 24 h from admission are related to an increased risk of adverse outcomes.	**Recommendation 3.2** We recommend against delaying appendectomy for acute appendicitis needing surgery beyond 24 h from the admission [QoE: Moderate; Strength of recommendation: Strong; 1B].
	**Statement 3.3** Appendectomy performed within the first 24 h from presentation in the case of uncomplicated appendicitis is not associated with an increased risk of perforation or adverse outcomes. Early appendectomy is the best management in complicated appendicitis.	**Recommendation 3.3** We suggest against delaying appendectomy for pediatric patients with uncomplicated acute appendicitis needing surgery beyond 24 h from the admission. Early appendectomy within 8 h should be performed in case of complicated appendicitis [QoE: Low; Strength of Recommendation: Weak; 2C].
**4. Surgical treatment**	**Statement 4.1** Laparoscopic appendectomy offers significant advantages over open appendectomy in terms of less pain, lower incidence of surgical site infection, decreased length of hospital stay, earlier return to work, overall costs, and better quality of life scores.	**Recommendation 4.1** We recommend laparoscopic appendectomy as the preferred approach over open appendectomy for both uncomplicated and complicated acute appendicitis, where laparoscopic equipment and expertise are available [QoE: High; Strength of recommendation: Strong; 1A].
	**Statement 4.2** Laparoscopic appendectomy is associated with lower postoperative pain, lower incidence of SSI and higher quality of life in children.	**Recommendation 4.2** We recommend laparoscopic appendectomy should be preferred over open appendectomy in children where laparoscopic equipment and expertise are available [QoE: Moderate; Strength of recommendation: Strong; 1B].
	**Statement 4.3** Single-incision laparoscopic appendectomy is basically feasible, safe, and as effective as conventional three-port laparoscopic appendectomy, operative times are longer, requires higher doses of analgesia, and is associated with a higher incidence of wound infection.	**Recommendation 4.3** We recommend conventional three-port laparoscopic appendectomy over single-incision laparoscopic appendectomy, as the conventional laparoscopic approach is associated with shorter operative times, less postoperative pain, and lower incidence of wound infection [QoE: High; Strength of recommendation: Strong; 1A].
	**Statement 4.4** In children with acute appendicitis, the single incision/transumbilical extracorporeal laparoscopic-assisted technique is as safe as the laparoscopic three-port technique.	**Recommendation 4.4** In pediatric patients with acute appendicitis and favorable anatomy, we suggest performing single incision/transumbilical extracorporeal laparoscopic assisted appendectomy or traditional three-port laparoscopic appendectomy based on local skills and expertise [QoE: Low; Strength of recommendation: Weak; 2C].
	**Statement 4.5** Outpatient laparoscopic appendectomy for uncomplicated acute appendicitis is feasible and safe without any difference in morbidity and readmission rates. It is associated with potential benefits of earlier recovery after surgery and lower hospital and social costs.	**Recommendation 4.5** We suggest the adoption of outpatient laparoscopic appendectomy for uncomplicated appendicitis, provided that an ambulatory pathway with well-defined ERAS protocols and patient information/consent are locally established [QoE: Moderate; Strength of recommendation: Weak; 2B].
	**Statement 4.6** Laparoscopic appendectomy seems to show relevant advantages compared to open appendectomy in obese adult patients, older patients, and patients with comorbidities. Laparoscopic appendectomy is associated with reduced mortality, reduced overall morbidity, reduced superficial wound infections, shorter operating times and postoperative length of hospital stay in such patients.	**Recommendation 4.6** We suggest laparoscopic appendectomy in obese patients, older patients and patients with high peri- and postoperative risk factors [QoE: Moderate; Strength of recommendation: Weak; 2B].
	**Statement 4.7** Laparoscopic appendectomy during pregnancy is safe in terms of risk of fetal loss and preterm delivery and it is preferable to open surgery as associated to shorter length of hospital stay and lower incidence of surgical site infection.	**Recommendation 4.7** We suggest laparoscopic appendectomy should be preferred to open appendectomy in pregnant patients when surgery is indicated. Laparoscopy is technically safe and feasible during pregnancy where expertise of laparoscopy is available [QoE: Moderate; Strength of recommendation: Weak; 2B].
	**Statement 4.8** Peritoneal irrigation does not have any advantage over suction alone in complicated appendicitis in both adults and children. The performance of irrigation during laparoscopic appendectomy does not seem to prevent the development of IAA and wound infections in neither adults nor pediatric patients.	**Recommendation 4.8** We recommend performing suction alone in complicated appendicitis patients with intra-abdominal collections undergoing laparoscopic appendectomy [QoE: Moderate; Strength of recommendation: Strong; 1B].
	**Statement 4.9** There are no clinical differences in outcomes, length of hospital stay and complications rates between the different techniques described for mesentery dissection (monopolar electrocoagulation, bipolar energy, metal clips, endoloops, LigaSure, Harmonic Scalpel, etc.).	**Recommendation 4.9** We suggest the use of monopolar electrocoagulation and bipolar energy as they are the most cost-effective techniques, whereas other energy devices can be used depending on the intra-operative judgment of the surgeon and resources available [QoE: Moderate; Strength of recommendation: Weak; 2B].
	**Statement 4.10** There are no clinical advantages in the use of endostaplers over endoloops for stump closure for both adults and children in either simple or complicated appendicitis, except for a lower incidence of wound infection when using endostaplers in children with uncomplicated appendicitis. Polymeric clips may be the cheapest and easiest method (with shorter operative times) for stump closure in uncomplicated appendicitis.	**Recommendation 4.10** We recommend the use of endoloops/suture ligation or polymeric clips for stump closure for both adults and children in either uncomplicated or complicated appendicitis, whereas endostaplers may be used when dealing with complicated cases depending on the intra-operative judgment of the surgeon and resources available [QoE: Moderate; Strength of recommendation: Strong; 1B].
	**Statement 4.11** Simple ligation should be preferred to stump inversion, either in open or laparoscopic surgery, as the major morbidity and infectious complications are similar. Simple ligation is associated with shorter operative times, less postoperative ileus and quicker recovery.	**Recommendation 4.11** We recommend simple ligation over stump inversion either in open and laparoscopic appendectomy [QoE: High; Strength of recommendation: Strong; 1A].
	**Statement 4.12** In adult patients, the use of drains after appendectomy for perforated appendicitis and abscess/peritonitis should be discouraged. Drains are of no benefit in preventing intra-abdominal abscess and lead to longer length of hospitalization and there is also low-quality evidence of increased 30-day morbidity and mortality rates in patients in the drain group.	**Recommendation 4.12** We recommend against the use of drains following appendectomy for complicated appendicitis in adult patients [QoE: Moderate; Strength of recommendation: Strong; 1B].
	**Statement 4.13** The prophylactic use of abdominal drainage after laparoscopic appendectomy for perforated appendicitis in children does not prevent postoperative complications and may be associated with negative outcomes.	**Recommendation 4.13** We suggest against the prophylactic use of abdominal drainage after laparoscopic appendectomy for complicated appendicitis in children [QoE: Low; Strength of recommendation: Weak; 2C].
	**Statement 4.14** The use of wound ring protectors shows some evidence of surgical site infection reduction in open appendectomy, especially in case of complicated appendicitis with contaminated/dirty wounds.	**Recommendation 4.14** We recommend wound ring protectors in open appendectomy to decrease the risk of SSI [QoE: Moderate; Strength of recommendation: Strong; 1B].
	**Statement 4.15** Delayed primary skin closure increases the length of hospital stay and overall costs in open appendectomies with contaminated/dirty wounds and does not reduce the risk of SSI. Subcuticular suture seems preferable in open appendectomy for acute appendicitis as it is associated with lower risk of complications (surgical site infection/abscess and seroma) and lower costs.	**Recommendation 4.15** We recommend primary skin closure with a unique absorbable intradermal suture for open appendectomy wounds [QoE: Moderate; Strength of recommendation: Weak; 2B].
**5. Intra-operative grading of acute appendicitis**	**Statement 5.1** The incidence of unexpected findings in appendectomy specimens is low. The intra-operative diagnosis alone is insufficient for identifying unexpected disease. From the currently available evidence, routine histopathology is necessary.	**Recommendation 5.1** We recommend routine histopathology after appendectomy [QoE: Moderate; Strength of recommendation: Strong; 1B].
	**Statement 5.2** Operative findings and intra-operative grading seem to correlate better than histopathology with morbidity, overall outcomes, and costs, both in adults and children. Intra-operative grading systems can help the identification of homogeneous groups of patients, determining optimal postoperative management according to the grade of the disease and ultimately improve the utilization of resources.	**Recommendation 5.2** We suggest the routine adoption of an intra-operative grading system for acute appendicitis (e.g., WSES 2015 grading score or AAST EGS grading score) based on clinical, imaging and operative findings [QoE: Moderate; Strength of recommendation: Weak; 2B].
	**Statement 5.3** Surgeon’s macroscopic judgment of early grades of acute appendicitis is inaccurate and highly variable. The variability in the intra-operative classification of appendicitis influences the decision to prescribe postoperative antibiotics and should be therefore prevented/avoided.	**Recommendation 5.3** We suggest appendix removal if the appendix appears “normal” during surgery and no other disease is found in symptomatic patients [QoE: Low; Strength of recommendation: Weak; 2C].
**6. Management of perforated appendicitis with phlegmon or abscess**	**Statement 6.1** Non-operative management is a reasonable first-line treatment for appendicitis with phlegmon or abscess. Percutaneous drainage as an adjunct to antibiotics, if accessible, could be beneficial, although there is a lack of evidence for its use on a routine basis. Laparoscopic surgery in experienced hands is a safe and feasible first-line treatment for appendiceal abscess, being associated with fewer readmissions and fewer additional interventions than conservative treatment, with a comparable hospital stay.	**Recommendation 6.1** We suggest non-operative management with antibiotics and—if available—percutaneous drainage for complicated appendicitis with periappendicular abscess, in settings where laparoscopic expertise is not available [QoE: Moderate; Strength of recommendation: Weak; 2B].
	**Statement 6.2** Operative management of acute appendicitis with phlegmon or abscess is a safe alternative to non-operative management in experienced hands, and may be associated with shorter LOS, reduced need for readmissions and fewer additional interventions than conservative treatment.	**Recommendation 6.2** We suggest the laparoscopic approach as treatment of choice for patients with complicated appendicitis with phlegmon or abscess where advanced laparoscopic expertise is available, with a low threshold for conversion. [QoE: Moderate; Strength of recommendation: Weak; 2B].
	**Statement 6.3** The reported rate of recurrence after non-surgical treatment for perforated AA and phlegmon ranges from 12% to 24%. Interval appendectomy and repeated NOM in case of recurrence of appendiceal phlegmon are associated with similar morbidity. However, elective interval appendectomy is related to additional operative costs to prevent recurrence in only one of eight patients, such as not to justify the routine performance of appendectomy.	**Recommendation 6.3** We recommend against routine interval appendectomy after NOM for complicated appendicitis in young adults (< 40 years old) and children. Interval appendectomy is recommended for those patients with recurrent symptoms [QoE: Moderate; Strength of recommendation: Strong; 1B].
	**Statement 6.4** The incidence of appendicular neoplasms is high (3–17%) in adult patients ≥ 40 years old) with complicated appendicitis.	**Recommendation 6.4** We suggest both colonic screening with colonoscopy and interval full-dose contrast-enhanced CT scan for patients with appendicitis treated non-operatively if ≥ 40 years old [QoE: Low; Strength of recommendation: Weak; 2C].
**7. Perioperative antibiotic therapy**	**Statement 7.1** A single dose of broad-spectrum antibiotics given preoperatively (from 0 to 60 min before the surgical skin incision) has been shown to be effective in decreasing wound infection and postoperative intra-abdominal abscess, regardless of the degree of inflammation of the removed appendix.	**Recommendation 7.1** We recommend a single preoperative dose of broad-spectrum antibiotics in patients with acute appendicitis undergoing appendectomy. We recommend against postoperative antibiotics for patients with uncomplicated appendicitis [QoE: High; Strength of recommendation: Strong; 1A].
	**Statement 7.2** In patients with complicated acute appendicitis, postoperative broad-spectrum antibiotics are suggested, especially if complete source control has not been achieved. For adult patients deemed to require them, discontinuation of antibiotics after 24 h seems safe and is associated with shorter length of hospital stay and lower costs. In patients with intra-abdominal infections who had undergone an adequate source control, the outcomes after fixed-duration antibiotic therapy (approximately 3–5 days) are similar to those after a longer course of antibiotics.	**Recommendation 7.2** We recommend against prolonging antibiotics longer than 3–5 days postoperatively in case of complicated appendicitis with adequate source-control [QoE: High; Strength of recommendation: Strong; 1A].
	**Statement 7.3** Administering postoperative antibiotics orally in children with complicated appendicitis for periods shorter than 7 days postoperatively seems to be safe and it is not associated with increased risk of complications. Early transition to oral antibiotics is safe, effective, and cost-efficient in the treatment of complicated appendicitis in the child.	**Recommendation 7.3** We recommend early switch (after 48 h) to oral administration of postoperative antibiotics in children with complicated appendicitis, with an overall length of therapy shorter than 7 days [QoE: Moderate; Strength of recommendation: Strong; 1B].
	**Statement 7.4** Postoperative antibiotics after appendectomy for uncomplicated acute appendicitis in children seems to have no role in reducing the rate of surgical site infection.	**Recommendation 7.4** In pediatric patients operated for uncomplicated acute appendicitis, we suggest against using postoperative antibiotic therapy [QoE: Low; Strength of recommendation: Weak; 2C].

### Topic 1: Diagnosis

#### Q.1.1: What is the value of clinical scoring systems in the management of adult patients with suspected appendicitis? Can they be used as basis for a structured management?

Risk stratification of patients with suspected AA by clinical scoring systems could guide decision-making to reduce admissions, optimize the utility of diagnostic imaging, and prevent negative surgical explorations. Clinical scores alone seem sufficiently sensitive to identify low-risk patients and decrease the need for imaging and negative surgical explorations (such as diagnostic laparoscopy) in patients with suspected AA.

The RCT by Andersson et al. demonstrated that, in low-risk patients, the use of an AIR (Appendicitis Inflammatory Response) score-based algorithm resulted in less imaging (19.2% vs 34.5%, *P* < 0.001), fewer admissions (29.5% vs 42.8%, *P* < 0.001), fewer negative explorations (1.6% vs 3.2%, *P* = 0.030), and fewer surgical operations for non-perforated AA (6.8% vs 9.7%, *P* = 0.034). Intermediate-risk patients randomized to the imaging and observation strategies had the same proportion of negative appendectomies (6.4% vs 6.7%, *P* = 0.884), number of hospital admissions, rates of perforation, and length of hospital stay, but routine imaging was associated with an increased proportion of patients treated for AA (53.4% vs 46.3%, *P* = 0.020) [29].

Among the many available clinical prediction models for the diagnosis of AA, the AIR score appears to be the best performer and most pragmatic. The review by Kularatna et al. recently summarized the results from validation studies, showing that the overall best performer in terms of sensitivity (92%) and specificity (63%) is the AIR score [30].

Although the Alvarado score is not sufficiently specific in diagnosing AA, a cutoff score of < 5 is sufficiently sensitive to exclude AA (sensitivity of 99%). The Alvarado score could, therefore, be used to reduce emergency department length of stay and radiation exposure in patients with suspected AA. This is confirmed by a large retrospective cohort study that found 100% of males with Alvarado score of 9 or greater, and 100% of females with an Alvarado score of 10 had AA confirmed by surgical pathology. Conversely, 5% or less of female patients with an Alvarado score of 2 or less and 0% of male patients with an Alvarado score of 1 or less were diagnosed with AA at surgery [31].

However, the Alvarado score is not able to differentiate complicated from uncomplicated AA in elderly patients and seems less sensitive in HIV+ patients [32, 33].

The RIPASA (Raja Isteri Pengiran Anak Saleha Appendicitis) score has shown to achieve better sensitivity and specificity than the Alvarado score in Asian and Middle Eastern population. Malik et al. recently published the first study evaluating the utility of the RIPASA score in predicting AA in a Western population. At a value of 7.5 (a cut of score suggestive of AA in the Eastern population), the RIPASA demonstrated reasonable sensitivity (85.39%), specificity (69.86%), positive predictive value (84.06%), negative predictive value (72.86%), and diagnostic accuracy (80%) in Irish patients with suspected AA and was more accurate than the Alvarado score [34].

The Adult Appendicitis Score (AAS) stratifies patients into three groups: high, intermediate, and low risk of AA. The score has been shown to be a reliable tool for stratification of patients into selective imaging, which results in a low negative appendectomy rate. In a prospective study enrolling 829 adults presenting with clinical suspicion of AA, 58% of patients with histologically confirmed AA had score value at least 16 and were classified as high probability group with 93% specificity. Patients with a score below 11 were classified as low probability of AA. Only 4% of patients with AA had a score below 11, and none of them had complicated AA. In contrast, 54% of non-AA patients had a score below 11. The area under ROC curve was significantly larger with the new score 0.882 compared with AUC of Alvarado score 0.790 and AIR score 0.810 [11].

In the validation study by Sammalkorpi et al., the AAS score stratified 49% of all AA patients into a high-risk group with the specificity of 93.3%, whereas in the low-risk group the prevalence of AA was 7%. The same study group demonstrated that diagnostic imaging has limited value in patients with a low probability of AA according to the AAS [35].

Tan et al. recently performed a prospective data collection on 350 consecutive patients with suspected AA for whom the Alvarado score for each patient was scored at admission and correlated with eventual histology and CT findings. The positive likelihood ratio of disease was significantly greater than 1 only in patients with an Alvarado score of 4 and above. An Alvarado score of 7 and above in males and 9 and above in females had a positive likelihood ratio comparable to that of CT scan [36].

Nearly all clinical signs and symptoms, as isolated parameters, do not significantly discriminate between those pregnant women with and without AA [37–39].

Of 15 validated risk prediction models taken into consideration in a recently published study enrolling 5345 patients with right iliac fossa pain across 154 UK hospitals, the AAS performed best for women (cutoff score 8 or less, specificity 63.1%, failure rate 3.7%), whereas the AIR score performed best for men (cutoff score 2 or less, specificity 24.7%, failure rate 2.4%) [40].

The Alvarado score can be higher in pregnant women due to the higher WBC values and the frequency of nausea and vomiting, especially during the first trimester, implicating lower accuracy compared to the non-pregnant population. Studies show Alvarado score (cutoff 7 points) sensitivity of 78.9% and specificity of 80.0% in pregnant patients [41, 42]. The RIPASA score has a specificity (cutoff 7.5 points) of 96%, but the score should be validated in larger studies. There are no studies of the Alvarado score discriminating between uncomplicated and complicated AA during pregnancy.

The preoperative distinction between uncomplicated and complicated AA is challenging. Recently, prediction models based on temperature, CRP, presence of free fluids on ultrasound, and diameter of the appendix have been shown to be useful for the identification of “high-risk” patients for complicated AA. Atema et al. found that, with the use of scoring systems combining clinical and imaging features, 95% of the patients deemed to have uncomplicated AA were correctly identified [43].

**Statement 1.1** Establishing the diagnosis of acute appendicitis based on clinical presentation and physical examination may be challenging. As the value of individual clinical variables to determine the likelihood of acute appendicitis in a patient is low, a tailored individualized approach is recommended, depending on disease probability, sex, and age of the patient. **Recommendation 1.1** We recommend to adopt a tailored individualized diagnostic approach for stratifying the risk and disease probability and planning an appropriate stepwise diagnostic pathway in patients with suspected acute appendicitis, depending on age, sex, and clinical signs and symptoms of the patient [QoE: Moderate; Strength of recommendation: Strong; 1B].

**Statement 1.2** Clinical scores alone, e.g., Alvarado score, AIR score, and the new Adult Appendicitis Score are sufficiently sensitive to exclude acute appendicitis, accurately identifying low-risk patients and decreasing the need for imaging and the negative appendectomy rates in such patients. **Recommendation 1.2.1** We recommend the use of clinical scores to exclude acute appendicitis and identify intermediate-risk patients needing of imaging diagnostics [QoE: High; Strength of recommendation: Strong; 1A]. **Recommendation 1.2.2** We suggest not making the diagnosis of acute appendicitis in pregnant patients on symptoms and signs only. Laboratory tests and inflammatory serum parameters (e.g., CRP) should always be requested [QoE: Very Low; Strength of recommendation: Weak; 2C].

**Statement 1.3** The Alvarado score is not sufficiently specific in diagnosing acute appendicitis in adults, seems unreliable in differentiating complicated from uncomplicated acute appendicitis in elderly patients, and is less sensitive in patients with HIV. **Recommendation 1.3** We suggest against the use of Alvarado score to positively confirm the clinical suspicion of acute appendicitis in adults [QoE: Moderate; Strength of recommendation: Weak; 2B].

**Statement 1.4** The AIR score and the AAS score seem currently to be the best performing clinical prediction scores and have the highest discriminating power in adults with suspected acute appendicitis. The AIR and AAS scores decrease negative appendectomy rates in low-risk groups and reduce the need for imaging studies and hospital admissions in both low- and intermediate-risk groups. **Recommendation 1.4** We recommend the use of AIR score and AAS score as clinical predictors of acute appendicitis [QoE: High; Strength of recommendation: Strong; 1A].

#### Q.1.2: In pediatric patients with suspected acute appendicitis could the diagnosis be based only on clinical scores?

AA is the most common surgical emergency in children, but early diagnosis of AA remains challenging due to atypical clinical features and the difficulty of obtaining a reliable history and physical examination. Several clinical scoring systems have been developed, the two most popular for use in children being the Alvarado score and Samuel’s Pediatric Appendicitis Score (PAS).

PAS includes similar clinical findings to the Alvarado score in addition to a sign more relevant in children: right lower quadrant pain with coughing, hopping, or percussion. Several studies comparing the PAS with the Alvarado score have validated its use in pediatric patients. However, in a systematic review by Kulik et al. both scores failed to meet the performance benchmarks of CRP (C-reactive protein). On average, the PAS would over-diagnose AA by 35%, and the Alvarado score would do so by 32% [44].

If we consider patients of preschool age, AA often presents with atypical features, more rapid progression, and higher incidence of complications. This age group is more likely to have lower PAS and Alvarado score than those of school-aged children [45]. This is the reason why Macco et al. retrospectively analyzed data from 747 children (mean age of 11 years) suspected of AA to evaluate the predictive value of the Alvarado score and PAS compared with the AIR score, which includes fewer symptoms than the Alvarado score and PAS, but adds the CRP value and allows for different severity levels of rebound pain, leukocytosis, CRP, and polymorphonucleocytes. The study showed that the AIR had the highest discriminating power and outperformed the other two scores in predicting AA in children [46].

The use of PAS seems to be useful to rule out or in AA in pediatric female patients. A retrospective observational study demonstrated that at a cutoff of ≥ 8, the PAS showed a specificity of 89% for adolescent females and 78% for all other patients, although the specificities did not differ at a cutoff of ≥ 7. At both cutoffs, the positive predictive values were poor in both groups. At a cutoff of ≥ 3, the PAS showed similar sensitivities in both groups [47]**.**

Recently, the new Pediatric Appendicitis Laboratory Score (PALabS) including clinical signs, leucocyte and neutrophil counts, CRP, and calprotectin levels has been shown to accurately predict which children are at low risk of AA and could be safely managed with close observation. A PALabS ≤ 6 has a sensitivity of 99.2%, a negative predictive value of 97.6%, and a negative likelihood ratio of 0.03 [48].

The preoperative clinical scoring system to distinguish perforation risk with pediatric AA proposed by Bonadio et al., based on the duration of symptoms (> 1 day), fever (> 38.0 C), and WBC absolute count (> 13,000/mm^3^), resulted in a multivariate ROC curve of 89% for perforation (*P* < 0.001), and the risk for perforation was additive with each additional predictive variable exceeding its threshold value, linearly increasing from 7% with no variable present to 85% when all 3 variables are present [49].

In assessing if the clinical scores can predict disease severity and the occurrence of complications, a retrospective study on the Alvarado score validity in pediatric patients showed that a higher median score was found in patients who suffered postoperative complications. The eight items in the scoring system were analyzed for their sensitivities. Fever, right lower quadrant tenderness, and neutrophilia were found to be the three most sensitive markers in predicting complicated AA (88.6%, 82.3%, and 79.7%). On the other hand, rebound tenderness was found to have the highest positive predictive value (65%) among the eight items to predict complicated AA [50].

**Statement 1.5** In pediatric patients with suspected acute appendicitis, the Alvarado score and Pediatric Appendicitis Score are useful tools in excluding acute appendicitis. **Recommendation 1.5** In pediatric patients with suspected acute appendicitis, we suggest against making a diagnosis based on clinical scores alone [QoE: Low; Strength of recommendation: Weak: 2C].

#### Q.1.3: What is the role of serum biomarkers in evaluating adult patients presenting with clinical features evocative of acute appendicitis?

The diagnostic accuracy of several biomarker panels has been prospectively validated, showing high sensitivity and negative predictive values for AA in large cohorts of patients with right iliac fossa pain, thereby potentially reducing the dependence on CT for the evaluation of possible AA [51].

The diagnostic value of baseline and early change of CRP concentrations has been evaluated separately or in combination with the modified Alvarado score in patients with clinically suspected AA in the prospective observational study by Msolli et al. Early change of CRP had a moderate diagnostic value in patients with suspected AA, and even combining CRP values to the modified Alvarado score did not improve diagnostic accuracy [52]. Recently, ischemia-modified albumin (IMA) levels have been used to determine the prediction of severity in AA patients. Kilic et al. found a strong positive correlation between IMA levels and CT findings in distinguishing gangrenous/perforated AA from uncomplicated AA [53]. A combination of clinical parameters, laboratory tests, and US may significantly improve diagnostic sensitivity and specificity and eventually replace the need for CT scan in both adults and children [54].

**Statement 1.6** Biochemical markers represent a promising reliable diagnostic tool for the identification of both negative cases or complicated acute appendicitis in adults. However, further high-quality evidence is needed [QoE: Low; No recommendation].

#### Q.1.4: What is the role of serum biomarkers in evaluating pediatric patients presenting clinical features highly suggestive of acute appendicitis?

In pediatric patients, routine diagnostic laboratory workup for suspected AA should include WBC, the differential with the calculation of the absolute neutrophil count (ANC), CRP, and urinalysis.

Although not widely available, the addition of procalcitonin and calprotectin to the above tests may significantly improve diagnostic discrimination [55].

Biomarkers have also been shown to be useful when used in association with the systematic adoption of scoring systems, as the addition of negative biomarker test results to patients with a moderate risk of AA based on the Pediatric Appendicitis Score (PAS) can safely reclassify many patients to a low-risk group. This may allow surgeons to provide more conservative management in patients with suspected AA and decrease unnecessary resource utilization [56].

Zouari et al. highlighted the value of CRP ≥ 10 mg/L as a strong predictor of AA in children < 6 years old [57].

Yu et al. reported that PCT had little value in diagnosing AA, with lower diagnostic accuracy than CRP and WBC, but a greater diagnostic value in identifying complicated AA [58]. In a recent meta-analysis, it was confirmed that PCT was more accurate in diagnosing complicated AA, with a pooled sensitivity of 0.89 (95% CI 0.84–0.93), specificity of 0.90 (95% CI 0.86–0.94), and diagnostic odds ratio of 76.73 (95% CI 21.6–272.9) [59].

Zani et al. retrospectively analyzed data from 1197 children admitted for AA and reported that patients with complicated AA had higher CRP and WBC levels than normal patients and those with uncomplicated AA. The authors found a CRP > 40 mg/L in 58% of patients with complicated AA and 37% of patients with uncomplicated AA, and WBC > 15 × 10^9^/L in 58% of patients with complicated AA and 43% of patients with uncomplicated AA [60].

One recent study identified a panel of biomarkers, the APPY1 test, consisting of WBC, CRP, and myeloid-related protein 8/14 levels that have the potential to identify, with great accuracy, children and adolescents with abdominal pain who are at low risk for AA. The biomarker panel exhibited a sensitivity of 97.1%, a negative predictive value of 97.4%, and a negative likelihood ratio of 0.08, with a specificity of 37.9% for AA [51].

Benito et al. prospectively evaluated the usefulness of WBC and ANC and other inflammatory markers such as CRP, procalcitonin, calprotectin, and the APPY1 test panel of biomarkers, to identify children with abdominal pain at low risk for AA. The APPY1 test panel showed the highest discriminatory power, with a sensitivity of 97.8, negative predictive value of 95.1, negative likelihood ratio of 0.06, and specificity of 40.6. In the multivariate analysis, only the APPY1 test and ANC > 7500/mL were significant risk factors for AA [55]**.**

More recently the Appendictis-PEdiatric score (APPE) was developed with the aim of identifying the risk of AA. Patients with an APPE score ≤ 8 were at low risk of AA (sensitivity 94%); those with a score ≥ 15 were at high risk for AA (specificity 93%). Those between 8 and 15 were defined at intermediate-risk [61].

A number of prospective studies of children were conducted to find urinary biomarkers for AA, such as leucine-rich α-2-glycoprotein (LRG), not to be used alone but combined with PAS and routine blood tests. LRG in conjunction with PAS showed 95% sensitivity, 90% specificity, 91% positive predictive value, and 95% negative predictive value for AA in children [62].

Among the new laboratory biomarkers developed, the Appendicitis Urinary Biomarker (AuB—leucine-rich alpha-2-glycoprotein) appears promising as a diagnostic tool for excluding AA in children, without the need for blood sampling (negative predictive value 97.6%) [63].

**Statement 1.7** White blood cell count, the differential with the calculation of the absolute neutrophil count, and the CRP are useful lab tests in predicting acute appendicitis in children; moreover, CRP level on admission ≥ 10 mg/L and leucocytosis ≥ 16,000/mL are strong predictive factors for appendicitis in pediatric patients. **Recommendation 1.6.1** In evaluating children with suspected appendicitis, we recommend to request routinely laboratory tests and serum inflammatory biomarkers [QoE: Very Low; Strength of recommendation: Strong: 1D]. **Recommendation 1.6.2** In pediatric patients with suspected acute appendicitis, we suggest adopting both biomarker tests and scores in order to predict the severity of the inflammation and the need for imaging investigation [QoE: Very Low; Strength of recommendation: Weak: 2D].

#### Q.1.5: What is the optimum pathway for imaging in adult patients with suspected acute appendicitis?

Estimating pre-image likelihood of AA is important in tailoring the diagnostic workup and using scoring systems to guide imaging can be helpful: low-risk adult patients according to the AIR/Alvarado scores could be discharged with appropriate safety netting, whereas high-risk patients are likely to require surgery rather than diagnostic imaging. Intermediate-risk patients are likely to benefit from systematic diagnostic imaging [64]. A positive US would lead to a discussion of appendectomy and a negative test to either CT or further clinical observation with repeated US. A conditional CT strategy, where CT is performed after the negative US, is preferable, as it reduces the number of CT scans by 50% and will correctly identify as many patients with AA as an immediate CT strategy.

Point-of-care ultrasonography (POCUS) has proven to be a valuable diagnostic tool in diagnosing AA and has a positive impact on clinical decision-making. Overall sensitivity and specificity of US is 76% and 95% and for CT is 99% and 84% respectively [65].

The meta-analysis by Matthew Fields et al. found that the sensitivity and specificity for POCUS in diagnosing AA were 91% and 97%, respectively. The positive and negative predictive values were 91% and 94%, respectively [66]. US reliability for the diagnosis of AA can be improved through standardized results reporting. In the study by Sola et al., following the adoption of a diagnostic algorithm that prioritized US over CT and encompassed standardized templates, the frequency of indeterminate results decreased from 44.3% to 13.1% and positive results increased from 46.4% to 66.1% in patients with AA [67].

Recent studies from the Finnish group led by Salminen demonstrated that the diagnostic accuracy of contrast-enhanced low-dose CT is not inferior to standard CT in diagnosing AA or distinguishing between uncomplicated and complicated AA, enabling significant radiation dose reduction. The OPTICAP randomized trial has shown that a low-dose protocol using intravenous contrast media was not inferior to the standard protocol in terms of diagnostic accuracy (79% accurate diagnosis in low-dose and 80% in standard CT by a primary radiologist) and accuracy to categorize AA severity (79% for both protocols). However, the mean radiation dose of low-dose CT was significantly lower compared with standard CT (3.33 and 4.44  mSv, respectively) [12]. The radiation dose of appendiceal CT for adolescents and young adults can be reduced to 2 mSv without impairing clinical outcomes and reducing the potential risk of exposure to ionizing radiation simultaneously [68]. The recently published Cochrane systematic review on CT scan for diagnosis of AA in adults identified 64 studies including 71 separate study populations with a total of 10280 participants (4583 with and 5697 without AA). Summary sensitivity of CT scan was 0.95, and summary specificity was 0.94. At the median prevalence of AA (0.43), the probability of having AA following a positive CT result was 0.92, and the probability of having AA following a negative CT result was 0.04. In subgroup analyses according to contrast enhancement, summary sensitivity was higher for CT with intravenous contrast (0.96), CT with rectal contrast (0.97), and CT with intravenous and oral contrast enhancement (0.96) than for non-enhanced CT (0.91). Summary sensitivity for low-dose CT (0.94) was similar to summary sensitivity for standard-dose or unspecified-dose CT (0.95). Summary specificity did not differ between low-dose and standard-dose or unspecified-dose CT [69].

The usefulness of CT for determining perforation in AA is limited [70]. Methods to improve precision in identifying patients with complicated AA should be explored, as these may help improve risk prediction for the failure of treatment with antibiotic therapy and guide patients and providers in shared decision-making for treatment options. In cases with equivocal CT features, repeat US and detection of specific US features (presence of non-compressibility and increased vascular flow of the appendix wall) can be used to discriminate AA from a normal appendix [71].

MRI has at least the same sensitivity and specificity as CT and, although has higher costs and issues around availability in many centers, should be preferred over CT as a first-line imaging study in pregnant women.

The American College of Radiology Appropriateness Criteria for pregnant women recommend graded compression grayscale US as a preferred initial method in case of suspected AA. These criteria recommend MRI as a second-line imaging method in inconclusive cases, although MRI can be used as a first-line imaging modality if available [72]. Others also recommend MRI after non-visualization or inconclusive US [73]. Despite some excellent US accuracy findings, the main drawback of US is the rate of non-visualization, which goes from 34.1% up to 71% with positive AA on the pathology reports [74, 75]. Low US accuracy for the diagnosis of AA in pregnant patients beyond the 1st trimester of pregnancy is evident and 30% of pregnant women with suspected AA have potentially avoidable surgery. Given the low yield of US, second-line imaging should be considered in those cases with an inconclusive US before surgery. A high rate (8%) of false-negative US results are positive on MRI [73, 76].

From 2011, there are three meta-analyses reporting on the use of MRI for AA during pregnancy with the following results: sensitivity 90.5%, 94%, and 91.8%; specificity 98.6%, 97%, and 97.9%; positive predictive value 86.3%; and negative predictive value 99.0% [77, 78]. Unfortunately, non-visualization of the appendix is up to 30–43% in some single-center series [79–82]. The rate of non-visualization is higher during the 3rd trimester when the largest degree of anatomic distortion occurs due to the gravid uterus [81].

Although a negative or inconclusive MRI does not exclude AA during pregnancy, many authors suggest MRI as the gold standard in all female patients during their reproductive years, mostly because of its high specificity and sensitivity (100% and 89%, respectively) and the high negative (96–100%) and positive (83.3–100%) predictive values [73, 83, 84].

**Statement 1.8** Combination of US and clinical (e.g., AIR, AAS scores) parameters forming combined clinico-radiological scores may significantly improve diagnostic sensitivity and specificity and eventually replace the need for a CT scan in adult patients with suspected acute appendicitis. **Recommendation 1.7** We recommend the routine use of a combination of clinical parameters and US to improve diagnostic sensitivity and specificity and reduce the need for CT scan in the diagnosis of acute appendicitis. The use of imaging diagnostics is recommended in patients with suspected appendicitis after an initial assessment and risk stratification using clinical scores [QoE: Moderate; Strength of recommendation: Strong; 1B].

**Statement 1.9** Intermediate-risk classification identifies patients likely to benefit from observation and systematic diagnostic imaging. **Recommendation 1.8** We suggest proceeding with timely and systematic diagnostic imaging in patients with intermediate-risk of acute appendicitis [QoE: Moderate; Strength of recommendation: Weak; 2B].

**Statement 1.10** Patients with strong signs and symptoms and high risk of appendicitis according to AIR score/Alvarado score/AAS and younger than 40 years old may not require cross-sectional pre-operative imaging (i.e., CT scan). **Recommendation 1.9** We suggest that cross-sectional imaging (i.e., CT scan) for high-risk patients younger than 40 years old (AIR score 9–12, Alvarado score 9–10, and AAS ≥ 16) may be avoided before diagnostic +/− therapeutic laparoscopy [QoE: Moderate; Strength of recommendation: Weak; 2B].

*Comment:* This statement and recommendation has raised an intense debate among the panel of experts and consensus was difficult to reach, especially in view of the strong opinions from two parties: one advocating the need of routine imaging with CT scan for all high-risk patients before any surgery and the other advocating the value of the clinical scores and thorough clinical assessment and risk stratification as being enough for proceeding to diagnostic and therapeutic laparoscopy in the subset of patients younger than 40 years old and scoring high in all Alvarado, AIR, and AAS scores.

The results of the first round of the Delphi consensus modified the previous recommendation from 2016 guidelines (see graphs included as Supplementary Material files 2, 3, 4, 5 and 6) as follows: “We suggest appendectomy without pre-operative imaging for high-risk patients younger than 50 years old according to the AIR score”, 8.3% agreement; “We suggest diagnostic +/− therapeutic laparoscopy without pre-operative imaging for high-risk patients younger than 40 years old, AIR score 9–12, Alvarado score 9–10, and AAS ≥ 16”, 70.8% agreement; “Delete recommendation”, 20.8% agreement) were discussed in a further consensus due to the strong opposition by few of the expert panelists who were still not keen to accept the results of the first Delphi and the recommendation despite being already labeled as a weak recommendation (“suggestion” according to GRADE Criteria).

A further revision of the statement was proposed and a second round of Delphi was performed before endorsing the final recommendation “We suggest that cross-sectional imaging i.e. CT scan for high-risk patients younger than 40 years old, AIR score 9–12 and Alvarado score 9–10 and AAS ≥ 16 may be avoided before diagnostic +/− therapeutic laparoscopy” which obtained the 68.0% of agreement, whereas the statement “We suggest diagnostic +/− therapeutic laparoscopy without pre-operative imaging for high-risk patients younger than 40 years old and AIR score 9–12; Alvarado score 9–10; AAS ≥ 16” reached 26% and the option “delete the statement and recommendations reached 6%. Some authors also added that cross-sectional imaging, i.e., CT scan for high-risk patients younger than 40 years old may be skipped or imaging may be avoided at all, before diagnostic +/− therapeutic laparoscopy for young male patients. Some also emphasized that the responsible surgeon (not PGY1 trainee) should examine the patient prior to the decision for CT scanning and recommended a highly value-based surgical care. WSES supports this recommendation of a value-based surgical care and these further comments will be the ground for the next future editions of the guidelines, when hopefully further and stronger evidence will be available from the literature about this challenging subgroup of high-risk scoring patients. All the graphs reporting the results of the additional Delphi are reported within the Supplementary Material files 2, 3, 4, 5 and 6.

**Statement 1.11** POCUS (Point-of-care Ultrasound) is a reliable initial investigation with satisfactory sensitivity and specificity in diagnosing acute appendicitis, easing swift decision-making by the emergency physicians or surgeons. POCUS, if performed by an experienced operator, should be considered the most appropriate first-line diagnostic tool in both adults and children. **Recommendation 1.10** We recommend POCUS as the most appropriate first-line diagnostic tool in both adults and children, if an imaging investigation is indicated based on clinical assessment [QoE: Moderate; Strength of recommendation: Strong; 1B].

**Statement 1.12** When it is indicated, contrast-enhanced low-dose CT scan should be preferred over contrast-enhanced standard-dose CT scan. Diagnostic accuracy of contrast-enhanced low-dose CT is not inferior to standard CT in diagnosing AA or distinguishing between uncomplicated and complicated acute appendicitis and enables significant radiation dose reduction. **Recommendation 1.11** We recommend the use of contrast-enhanced low-dose CT scan over contrast-enhanced standard-dose CT scan in patients with suspected acute appendicitis and negative US findings [QoE: High; Strength of recommendation: Strong; 1A].

**Statement 1.13** In patients with normal investigations and symptoms unlikely to be acute appendicitis but which do not settle, cross-sectional imaging is recommended before surgery. Laparoscopy is recommended to establish/exclude the diagnosis of acute appendicitis and eventually treat the disease. **Recommendation 1.12** We recommend cross-sectional imaging before surgery for patients with normal investigations but non-resolving right iliac fossa pain. After negative imaging, initial non-operative treatment is appropriate. However, in patients with progressive or persistent pain, explorative laparoscopy is recommended to establish/exclude the diagnosis of acute appendicitis or alternative diagnoses [QoE: High; Strength of recommendation: Strong; 1A].

**Statement 1.14** MRI is sensitive and highly specific for the diagnosis of acute appendicitis during pregnancy. However, a negative or inconclusive MRI does not exclude appendicitis and surgery should be still considered if high clinical suspicion. **Recommendation 1.13.1** We suggest graded compression trans-abdominal ultrasound as the preferred initial imaging method for suspected acute appendicitis during pregnancy [QoE: Very Low; Strength of Recommendation: Weak; 2C]. **Recommendation 1.13.2** We suggest MRI in pregnant patients with suspected appendicitis, if this resource is available, after inconclusive US [QoE: Moderate; Strength of recommendation: Weak; 2B].

#### Q.1.6: What is the optimum pathway for imaging in pediatric patients with suspected acute appendicitis?

US is currently the recommended initial imaging study of choice for the diagnosis of AA in pediatric and young adult patients. US has been shown to have high diagnostic accuracy for AA as an initial imaging investigation and to reduce or obviate the need for further imaging without increased complications or unacceptable increases in length of stay [85].

However, the sensitivity and specificity of US for the diagnosis of pediatric AA varies across studies: it is well known that US is operator dependent and may be dependent on patient-specific factors, including BMI [86].

A retrospective study assessing the ability of US to identify complicated AA or an appendicolith showed that US has a high specificity and negative predictive value to exclude complicated AA and the presence of an appendicolith in children being considered for non-operative management of uncomplicated AA [87].

The study by Bachur et al. found that, among children with suspected AA, the use of US imaging has increased substantially (from 24.0% in 2010 to 35.3% in 2013), whereas the use of CT has decreased (from 21.4% in 2010 to 11.6% in 2013). However, important condition-specific quality measures, including the frequency of appendiceal perforation and readmissions, remained stable, and the proportion of negative appendectomy declined slightly [88].

The use of CT in the pediatric population can be reduced by using appropriate clinical and/or staged algorithm based on US/MRI implementation, with a sensitivity up to 98% and a specificity up to 97% and by applying imaging scoring system, such as the Appy-Score for reporting limited right lower quadrant US exams, that performs well for suspected pediatric AA [89–91].

A systematic literature review was performed to evaluate the effectiveness of abdominal US and abdominal CT in diagnosing AA in adult and pediatric patients. Data reported that for US, the calculated pooled values of sensitivity, specificity, positive predictive value, and negative predictive value were 86%, 94%, 100%, and 92%, respectively. For CT, the calculated pooled values of sensitivity, specificity, positive predictive value, and negative predictive value were 95%, 94%, 95%, and 99%, respectively. These results suggest that US is an effective first-line diagnostic tool for AA and that CT should be performed for patients with inconclusive ultrasonographic finding [92]. Recently, a meta-analysis was carried out to compare the accuracy of US, CT, and MRI for clinically suspected AA in children. The area under the receiver operator characteristics curve of MRI (0.995) was a little higher than that of US (0.987) and CT (0.982) but with no significant difference [93].

Lee et al. compared US and CT in terms of negative appendectomy rate and appendiceal perforation rate in adolescents and adults with suspected appendicitis to evaluate the diagnostic performance as preoperative imaging investigations with a propensity score method. This analysis reported that the use of US instead of CT may increase the negative appendectomy rate but does not significantly affect the rate of perforation [94].

A low dose CT, when indicated, can be an adequate method compared to US and standard dose CT in diagnosing AA in children in terms of sensitivity (95.5% vs 95.0% and 94.5%), specificity (94.9% vs 80.0% and 98.8%), positive-predictive value (96.4% vs 92.7%), and negative-predictive value (93.7% vs 85.7% and 91.3%) [95].

The diagnostic performance of staged algorithms involving US followed by conditional MRI imaging for the diagnostic workup of pediatric AA has proven to be high (98.2% sensitive and 97.1% specific) [90]. MRI is a feasible alternative to CT for secondary imaging in AA in children, and it can differentiate perforated from non-perforated AA with a high specificity [96].

MRI plays a role as an imaging investigation to avoid CT radiation dose in children with inconclusive US findings. Moore et al. reported sensitivity of 96.5%, specificity of 96.1%, positive predictive value of 92.0%, and negative predictive value of 98.3% for MRI [97].

In a prospective study conducted by Kinner et al., when the diagnostic accuracy of MRI was compared to CT, sensitivity and specificity were 85.9% and 93.8% for non-enhanced MRI, 93.6% and 94.3% for contrast-enhanced MRI, and 93.6% and 94.3% for CT [98].

However, the costs and the availability of MRI often prevent its use as the initial imaging investigation in cases of suspected AA.

As second-line imaging modalities after initial US for assessing AA in children and adults, repeated US, CT, and MRI showed comparable and high accuracy in children and adults. These three modalities may be valid as second-line imaging in a clinical imaging pathway for diagnosis of AA. In particular, pooled sensitivities and specificities of second-line US for the diagnosis of AA in children were 91.3% and 95.2%, respectively. Regarding second-line CT, the pooled sensitivities and specificities were 96.2% and 94.6%. Regarding second-line MRI, pooled sensitivities and specificities were 97.4% and 97.1% [99].

**Statement 1.15** The use of US in children is accurate and safe in terms of perforation rates, emergency department re-visits, and negative appendectomy rates. CT use may be decreased by using appropriate clinical and/or staged algorithm with US/MRI. MRI has at least the same sensitivity and specificity as CT and, although higher costs, should be preferred over CT as second-line imaging in children. **Recommendation 1.14.1** In pediatric patients with suspected appendicitis, we suggest the use of US as first-line imaging. In pediatric patients with inconclusive US, we suggest choosing the second-line imaging technique based on local availability and expertise, as there are currently no strong data to suggest a best diagnostic pathway due to a variety of options and dependence on local resources [QoE: Moderate; Strength of recommendation: Weak: 2B]. **Recommendation 1.14.2** Since in pediatric patients with equivocal CT finding the prevalence of true acute appendicitis is not negligible, we suggest against the routine use of CT as first-line imaging in children with right iliac fossa pain [QoE: Moderate; Strength of recommendation: Weak; 2B].

### Topic 2: Non-operative management of uncomplicated acute appendicitis

#### Q.2.1: Is non-operative management with or without antibiotics a safe and effective treatment option for adult patients with uncomplicated acute appendicitis?

Recent systematic reviews and meta-analyses of RCTs have concluded that the majority of patients with uncomplicated AA can be treated with an antibiotic-first approach [16, 18, 100].

The recent meta-analysis by Harnoss et al. reported a recurrence rate of symptoms within 1 year of 27.4% following antibiotic-first treatment. Taking into consideration any kind of post-interventional complication (including treatment failure), the complication-free treatment success rate of antibiotic therapy was significantly inferior to the rate after surgery (68.4 vs 89.8%). There is also evidence that NOM for uncomplicated AA does not statistically increase the perforation rate in adult patients receiving antibiotic treatment. NOM with antibiotics may fail during the primary hospitalization in about 8% of cases, and an additional 20% of patients might need a second hospitalization for recurrent AA within 1 year from the index admission [16, 17].

The success of the non-operative approach requires careful patient selection and exclusion of patients with gangrenous AA, abscesses, and diffuse peritonitis. Hansson et al. in their study on 581 patients with AA published in 2014 found that patients with assumed AA who fulfilled all criteria with CRP < 60 g/L, WBC < 12 × 10^9^/L, and age < 60 years had an 89% of chance of recovery with antibiotics without surgery [101]. In another recent study, patients with a longer duration of symptoms prior to admission (> 24 h) were more likely to have successful NOM. Other independent predictors of NOM success included lower temperature, imaging-confirmed uncomplicated AA with lower modified Alvarado score (< 4), and smaller diameter of the appendix [102].

In the APPAC randomized trial appendectomy resulted in an initial success rate of 99.6%. In the antibiotic group, 27.3% of patients underwent appendectomy within 1 year of initial presentation for AA. Of the 256 patients available for follow-up in the antibiotic group, 72.7% did not require surgery. Of the 70 patients randomized to antibiotic treatment who subsequently underwent appendectomy, 82.9% had uncomplicated AA, 10.0% had complicated AA, and 7.1% did not have AA but received appendectomy for suspected recurrence. There were no intra-abdominal abscesses or other major complications associated with delayed appendectomy in patients randomized to antibiotic treatment [103].

The 5-year follow-up results of the APPAC trial reported that, among patients who were initially treated with antibiotics, the likelihood of late recurrence was 39.1%. Only 2.3% of patients who had surgery for recurrent AA were diagnosed with complicated forms of the disease. The overall complication rate was significantly reduced in the antibiotic group compared to the appendectomy group (6.5% vs 24.4%). This long-term follow-up supports the feasibility of NOM with antibiotics as an alternative to surgery for uncomplicated AA [104]. Furthermore, patients receiving antibiotic therapy incur lower costs than those who had surgery [105].

The presence of an appendicolith has been identified as an independent prognostic risk factor for treatment failure in NOM of uncomplicated AA. When presenting together with AA, the presence of appendicoliths is associated with increased perforation risk. The recently published study by Mällinen et al. further corroborates the previous clinical hypothesis showing that the presence of an appendicolith is an independent predictive factor for both perforation and the failure of NOM of uncomplicated AA [106–108].

Case reports show that it may be possible to manage uncomplicated AA non-operatively (definitively or as a bridge therapy) during pregnancy [109, 110]**.** There is a single study, with 25% of pregnant patients with uncomplicated AA treated conservatively. The failure rate was 15%. Recurrence rate during the same pregnancy was 12% [111]. A small number of published cases had different antibiotic regimens which include different antibiotics or their combinations and different durations of initial intravenous administration with different duration of antibiotic continuation in the form of oral administration (3–7 days in total) [102, 111].

**Statement 2.1** The antibiotic-first strategy can be considered safe and effective in selected patients with uncomplicated acute appendicitis. Patients who wish to avoid surgery must be aware of a risk of recurrence of up to 39% after 5 years. Most recent data from meta-analyses of RCTs showed that NOM with antibiotics achieves a significantly lower overall complication rate at 5 years and shorter sick leave compared to surgery. **Recommendation 2.1.1** We recommend discussing NOM with antibiotics as a safe alternative to surgery in selected patients with uncomplicated acute appendicitis and absence of appendicolith, advising of the possibility of failure and misdiagnosing complicated appendicitis [QoE: High; Strength of Recommendation: Strong; 1A]. **Recommendation 2.1.2** We suggest against treating acute appendicitis non-operatively during pregnancy until further high-level evidence is available [QoE: Very Low; Strength of Recommendation: Weak; 2C].

#### Q.2.2: Is non-operative management with or without antibiotics a safe and effective treatment option for pediatric patients with uncomplicated acute appendicitis?

Less than 19% of children have a complicated acute appendicitis; hence, the majority of children with uncomplicated AA may be considered for either a non-operative or an operative management [112].

The antibiotic-first strategy appears effective as an initial treatment in 97% of children with uncomplicated AA (recurrence rate 14%), with NOM also leading to less morbidity, fewer disability days, and lower costs than surgery [113, 114].

A systematic review of all evidence available comparing appendectomy to NOM for uncomplicated AA in children included 13 studies, 4 of which were retrospective studies, 4 prospective cohort studies, 4 prospective non-randomized comparative trials, and 1 RCT. The initial success of the NOM groups ranged from 58 to 100%, with 0.1–31.8% recurrence at 1 year [115].

The meta-analysis by Huang et al. showed that antibiotics as the initial treatment for pediatric patients with uncomplicated AA may be feasible and effective without increasing the risk of complications. However, surgery is preferred for uncomplicated AA with the presence of an appendicolith as the failure rate in such cases is high [116].

The prospective trial by Mahida et al. reported that the failure rate of NOM with antibiotics in children affected by uncomplicated AA with appendicolith was high (60%) at a median follow-up of less than 5 months [117]. The presence of an appendicolith has also been associated with high failure rates in the reports published by Tanaka et al. (failure rate, 47%), Svensson et al. (failure rate, 60%), and Lee et al., concluding that patients with evidence of an appendicolith on imaging had an initial NOM failure rate of more than twice that of patients without an appendicolith [118–120].

Gorter et al. investigated the risk of complications following NOM and appendectomy for uncomplicated AA in a systematic review. Five studies (RCT and cohort studies) were analyzed, including 147 children (NOM) and 173 children (appendectomy) with 1-year follow-up. The percentage of children experiencing complications ranged from 0 to 13% for NOM versus 0–17% for appendectomy. NOM avoided an appendectomy in 62–81% of children after 1-year follow-up. The authors concluded that NOM can avoid an appendectomy in a large majority of children after 1-year follow-up but evidence was insufficient to suggest NOM in all children with uncomplicated AA [121].

In the meta-analysis by Kessler et al. NOM showed a reduced treatment efficacy (relative risk 0.77, 95% CI 0.71–0.84) and an increased readmission rate (relative risk 6.98, 95% CI 2.07–23.6), with a comparable rate of complications (relative risk 1.07, 95% CI 0.26–4.46). Exclusion of patients with appendicoliths improved treatment efficacy in conservatively treated patients. The authors concluded that NOM was associated with a higher readmission rate [122].

Considering these results, NOM can be suggested only for selected pediatric patients presenting with uncomplicated AA.

Minneci et al. conducted a prospective patient choice cohort study enrolling 102 patients aged 7 to 17 years and showed that the incidence of complicated AA was 2.7% in the NOM group and 12.3% in the appendectomy group. After 1 year, children managed nonoperatively had fewer disability days and lower appendicitis-related health care costs compared with those who underwent appendectomy [114].

**Statement 2.2** NOM for uncomplicated acute appendicitis in children is feasible, safe, and effective as initial treatment. However, the failure rate increases in the presence of appendicolith, and surgery is recommended in such cases. **Recommendation 2.2** We suggest discussing NOM with antibiotics as a safe and effective alternative to surgery in children with uncomplicated acute appendicitis in the absence of an appendicolith, advising of the possibility of failure and misdiagnosing complicated appendicitis [QoE: Moderate; Strength of recommendation: Weak; 2B].

#### Q.2.3: What is the best non-operative management of patients with uncomplicated acute appendicitis?

The implementation of treatment and follow-up protocols based on outpatient antibiotic management and new evidence indicating safety and feasibility of same-day laparoscopic appendectomy for uncomplicated AA may result in optimization of the resource used by reducing inpatient admissions and hospital costs for both NOM and surgical treatment in the future. Although the pilot trial by Talan et al. assessed the feasibility of antibiotics-first strategy including outpatient management (intravenous ertapenem greater than or equal to 48 h and oral cefdinir and metronidazole), the majority of RCTs published to date included 48 h minimum of inpatient administration of intravenous antibiotics, followed by oral antibiotics for a total length of 7–10 days [123].

The empiric antibiotic regimens for non-critically ill patients with community-acquired intra-abdominal infections as advised by the 2017 WSES guidelines are the following: Amoxicillin/clavulanate 1.2–2.2 g 6-hourly or ceftriazone 2 g 24-hourly + metronidazole 500 mg 6-hourly or cefotaxime 2 g 8-hourly + metronidazole 500 mg 6-hourly.

In patients with beta-lactam allergy: Ciprofloxacin 400 mg 8-hourly + metronidazole 500 mg 6-hourly or moxifloxacin 400 24-hourly. In patients at risk for infection with community-acquired ESBL-producing Enterobacteriacea: Ertapenem 1 g 24-hourly or tigecycline 100 mg initial dose, then 50 mg 12-hourly [124].

Currently, the APPAC II trial is running, with the aim to assess the safety and feasibility of per-oral antibiotic monotherapy compared with intravenous antibiotic therapy continued by per oral antibiotics in the treatment of uncomplicated AA. Early results of the APPAC II are expected to be published in 2020 [125].

The results of the RCT by Park et al. challenged the need for antibiotic therapy in uncomplicated AA and reported promising results regarding possible spontaneous resolution of uncomplicated AA with supportive care only. Analysis of the primary outcome measure indicated that treatment failure rates in patients presenting with CT-confirmed uncomplicated AA were similar among those receiving supportive care with either a non-antibiotic regimen or a 4-day course of antibiotics, with no difference in the rates of perforated AA between the two groups reported [126]. Whether recovery from uncomplicated AA is the result of antibiotic therapy or natural clinical remission, and so whether antibiotics are superior to simple supportive care remains to be established.

The APPAC III multicenter, double-blind, placebo-controlled, superiority RCT comparing antibiotic therapy with placebo in the treatment of CT scan-confirmed uncomplicated AA is now in its enrollment phase. This new RCT aims to evaluate the role of antibiotics in the resolution of CT-diagnosed uncomplicated AA by comparing antibiotic therapy with placebo to evaluate the role of antibiotic therapy in the resolution of the disease [127].

If future research demonstrates that antibiotics do not provide any advantage over observation alone in uncomplicated AA, this could have a major impact on reducing the use of antimicrobial agents, especially in this era of increasing antimicrobial resistance worldwide.

**Statement 2.3** Current evidence supports initial intravenous antibiotics with subsequent conversion to oral antibiotics until further evidence from ongoing RCT is available. **Recommendation 2.3** In the case of NOM, we recommend initial intravenous antibiotics with a subsequent switch to oral antibiotics based on patient's clinical conditions [QoE: Moderate; Strength of recommendation: Strong; 1B].

**Statement 2.4** Uncomplicated acute appendicitis may safely resolve spontaneously with similar treatment failure rates and shorter length of stay and costs compared with antibiotics. However, there is still limited data for the panel to express in favor of or against the symptomatic treatment without antibiotics [QoE: Moderate; No recommendation].

### Topic 3: Timing of appendectomy and in-hospital delay

#### Q.3.1: Does in-hospital delay increase the rate of complications or perforation for adult patients with uncomplicated acute appendicitis?

The theory hypothesizing that perforated AA might be a different disease entity from uncomplicated AA, rather than being the natural evolution of the disease, has some support in the recent meta-analysis by van Dijk et al., demonstrating that delaying appendectomy for up to 24 h after admission does not appear to be a risk factor for complicated AA, postoperative morbidity, or surgical-site infection. Pooled adjusted ORs revealed no significantly higher risk for complicated AA when appendicectomy was delayed for 7–12 or 13–24 h, and meta-analysis of unadjusted data supported these findings by yielding no increased risk for complicated AA or postoperative complications with a delay of 24–48 h [22].

Data from the American College of Surgeons NSQIP demonstrated similar outcomes of appendectomy for AA when the operation was performed on hospital day 1 or 2. Conversely, appendectomies performed on hospital day 3 had significantly worse outcomes, as demonstrated by increased 30-day mortality (0.6%) and all major postoperative complications (8%) in comparison with operations taking place on hospital day 1 (0.1%; 3.4%) or 2 (0.1%; 3.6%). Patients with decreased baseline physical status assessed by the ASA Physical Status class had the worst outcomes (1.5% mortality; 14% major complications) when an operation was delayed to hospital day 3. However, logistic regression revealed higher ASA Physical Status class and open operations as the only predictors of major complications [128].

In the study by Elniel et al., a significant increase in the likelihood of perforated AA occurred after 72 h of symptoms, when compared to 60–72 h. The authors argued that it may be reasonable to prioritize patients approaching 72 h of symptoms for operative management [129].

In a large retrospective series of pregnant women with suspected AA (75.9% with uncomplicated AA, 6.5% with complicated AA, and 17.6% with normal appendix), initial US was diagnostic in 57.9% of patients, whereas 55.8% of patients underwent a delayed repeat study. In this cohort, performing a delayed repeat US during a period of observation in those patients who remained otherwise equivocal increased the diagnostic yield of the US, whereas delaying surgery did not affect maternal or fetal safety. Such algorithm increased the diagnostic yield without increasing the proxies of maternal or fetal morbidity. There was no increased rate of perforated appendices in patients with delayed surgery. Still, the negative appendectomy rate was 17.7% [130].

**Statement 3.1** Short, in-hospital surgical delay up to 24 h is safe in uncomplicated acute appendicitis and does not increase complications and/or perforation rate in adults. Surgery for uncomplicated acute appendicitis can be planned for the next available list minimizing delay wherever possible (better patient comfort, etc.). Short, in-hospital delay with observation and repeated trans-abdominal US in pregnant patients with equivocal appendicitis is acceptable and does not seem to increase the risk of maternal and fetal adverse outcomes. **Recommendation 3.1** We recommend planning laparoscopic appendectomy for the next available operating list within 24 h in case of uncomplicated acute appendicitis, minimizing the delay wherever possible [QoE: Moderate; Strength of recommendation: Strong; 1B].

**Statement 3.2** Delaying appendectomy for uncomplicated acute appendicitis for up to 24 h after admission does not appear to be a risk factor for complicated appendicitis, postoperative surgical site infection, or morbidity. Conversely, appendectomies performed after 24 h from admission are related to increased risk of adverse outcomes. **Recommendation 3.2** We recommend against delaying appendectomy for acute appendicitis needing surgery beyond 24 h from the admission [QoE: Moderate; Strength of recommendation: Strong; 1B].

#### Q.3.2: Does in-hospital delay increase the rate of complications or perforation for pediatric patients with uncomplicated acute appendicitis?

In children appendectomy performed within the first 24 h from presentation is not associated with an increased risk of perforation or adverse outcomes [131]. Similarly, in the multivariate logistic regression analysis by Almstrom et al., increased time to surgery was not associated with increased risk of histopathologic perforation, and there was no association between the timing of surgery and postoperative wound infection, intra-abdominal abscess, reoperation, or readmission [132].

Data from NSQIP-Pediatrics demonstrated that a 16-h delay from emergency department presentation or a 12-h delay from hospital admission to appendectomy was not associated with an increased risk of SSI. Compared with patients who did not develop an SSI, patients who developed an SSI had similar times between emergency department triage and appendectomy (11.5  h vs 9.7  h, *P*  =  0.36) and similar times from admission to appendectomy (5.5  h vs 4.3  h, *P*  =  0.36). Independent risk factors for SSI were complicated AA, longer symptom duration, and presence of sepsis/septic shock [133].

Gurien et al. retrospectively analyzed data from 484 children who underwent appendectomy at 6, 8, and 12 h from admission for AA and reported a mean elapsed time from admission to theatre of 394 min. SSIs, appendiceal perforations, and small bowel obstructions were similar between early and delayed groups, and no statistically significant differences were found for SSIs in the non-perforated delayed versus immediate groups. Time from admission to theatre did not predict perforation, whereas WBC count at the time of admission was a significant predictor of perforation (OR 1.08; *P* < 0.001) [134].

Recently, the American Pediatric Surgical Association Outcomes and Evidence-Based Practice Committee developed recommendations regarding time to appendectomy for AA in children by a systematic review of the published articles between January 1, 1970, and November 3, 2016. The committee stated that appendectomy performed within the first 24 h from presentation is not associated with an increased risk of perforation or adverse outcomes [135].

Regarding complicated AA, some authors support initial antibiotics with delayed operation whereas others support immediate operation. Regarding complicated appendicitis, some authors support initial antibiotics with delayed operation whereas others support immediate operation. A population-level study with a 1-year follow-up period found that children undergoing late appendectomy were more likely to have a complication than those undergoing early appendectomy. These data support that early appendectomy is the best management in complicated AA [136].

**Statement 3.3** Appendectomy performed within the first 24 h from presentation in the case of uncomplicated appendicitis is not associated with an increased risk of perforation or adverse outcomes. Early appendectomy is the best management in complicated appendicitis. **Recommendation 3.3** We suggest against delaying appendectomy for pediatric patients with uncomplicated acute appendicitis needing surgery beyond 24 h from the admission. Early appendectomy within 8 h should be performed in case of complicated appendicitis [QoE: Low; Strength of Recommendation: Weak; 2C].

### Topic 4: Surgical treatment

#### Q.4.1: Does laparoscopic appendectomy confer superior outcomes compared with open appendectomy for adult patients with acute appendicitis?

Several systematic reviews of RCTs comparing laparoscopic appendectomy (LA) versus open appendectomy (OA) have reported that the laparoscopic approach for AA is often associated with longer operative times and higher operative costs, but it leads to less postoperative pain, shorter length of stay, and earlier return to work and physical activity [137]. LA lowers overall hospital and social costs [138], improves cosmesis, and significantly decreases postoperative complications, in particular SSI.

The 2018 updated Cochrane review on LA versus OA showed that, except for a higher rate of IAA (intra-abdominal abscess) after LA in adults, laparoscopic demonstrates advantages over OA in pain intensity on day one, SSI, length of hospital stay, and time until return to normal activity [139].

In the meta-review by Jaschinski et al. including nine systematic reviews and meta-analyses (all moderate to high quality), the pooled duration of surgery was 7.6 to 18.3 min shorter with OA. Pain scores on the first postoperative day were lower after LA in two out of three reviews. The risk of IAA was higher for LA in half of six meta-analyses, whereas the occurrence of SSI pooled by all reviews was lower after LA. LA shortened hospital stay from 0.16 to 1.13 days in seven out of eight meta-analyses [14].

The evidence regarding treatment effectiveness of LA versus OA in terms of postoperative IAA, however, changed over the last decade. The cumulative meta-analysis by Ukai et al. demonstrated that, of the 51 trials addressing IAA, trials published up to and including 2001 showed statistical significance in favor of OA. The effect size in favor of OA began to disappear after 2001, leading to an insignificant result with an overall cumulative OR of 1.32 (95% CI 0.84–2.10) when LA was compared with OA [140].

LA appears to have significant benefits with improved morbidity compared to OA in complicated AA as well, as demonstrated in the meta-analysis by Athanasiou et al. In the pooled analysis, LA had significantly less SSI, with reduced time to oral intake, and length of hospitalization. There was no significant difference in IAA rates. Operative time was longer during LA but did not reach statistical significance in the RCT subgroup analysis [141].

**Statement 4.1** Laparoscopic appendectomy offers significant advantages over open appendectomy in terms of less pain, lower incidence of surgical site infection, decreased length of hospital stay, earlier return to work, overall costs, and better quality of life scores. **Recommendation 4.1** We recommend laparoscopic appendectomy as the preferred approach over open appendectomy for both uncomplicated and complicated acute appendicitis, where laparoscopic equipment and expertise are available [QoE: High; Strength of recommendation: Strong; 1A].

#### Q.4.2: Does laparoscopic appendectomy confer superior outcomes compared with open appendectomy for pediatric patients with acute appendicitis?

The laparoscopic approach to AA seems to be safe and effective in children.

Zhang et al. conducted a meta-analysis of nine studies to compare the influence of different surgical procedures on perforated AA in the pediatric population and found that LA was associated with lower incidence of SSI and bowel obstruction, but the rate of IAA was higher than in OA [142].

Yu et al. conducted a meta-analysis of two RCTs and 14 retrospective cohort studies, showing that LA for complicated AA reduces the rate of SSIs (OR 0.28; 95% CI 0.25–0.31) without increasing the rate of postoperative IAA (OR 0.79; 95% CI 0.45–1.34). The results showed that the operating time in the LA group was longer than that of the OA groups (WMD 13.78, 95% CI 8.99–18.57), whereas the length of hospital stay in the LA groups was significantly shorter (WMD − 2.47, 95% CI − 3.75 to − 1.19), and the time to oral intake was shorter in the LA group than in the OA group (WMD − 0.88, 95% CI − 1.20 to − 0.55) [15].

**Statement 4.2** Laparoscopic appendectomy is associated with lower postoperative pain, lower incidence of SSI, and higher quality of life in children. **Recommendation 4.2** We recommend laparoscopic appendectomy should be preferred over open appendectomy in children where laparoscopic equipment and expertise are available [QoE: Moderate; Strength of recommendation: Strong; 1B].

#### Q.4.3: Does laparoscopic single-incision surgery confer any advantage over the three-trocar technique in performing laparoscopic appendectomy for adult patients with acute appendicitis?

Recent studies provide level 1a evidence that single-incision laparoscopic appendectomy (SILA) is as feasible, effective, and safe as the conventional three-port LA. High-level meta-analyses conducted in adults, although demonstrating no significant difference in the safety of SILA versus that of three-port LA, have not supported the application of SILA because of its significantly longer operative times and the higher doses of analgesia required compared with those for three-port LA [143]. A total of 8 RCTs published between 2012 and 2014 with a total of 995 patients were included in the meta-analysis by Aly et al. No significant differences between SILA and conventional three-port laparoscopic appendectomy (CLA) was found in terms of complication rates, postoperative ileus, length of hospital stay, return to work, or postoperative pain. CLA was significantly superior to SILA with reduced operating time (mean difference 5.81 [2.01, 9.62], *P* = 0.003) and conversion rates (OR 4.14 [1.93, 8.91], *P* = 0.0003). Conversely, SILA surgery had better wound cosmesis (mean difference 0.55 [0.33, 0.77], *P* = 0.00001) [144].

**Statement 4.3** Single-incision laparoscopic appendectomy is basically feasible, safe, and as effective as conventional three-port laparoscopic appendectomy, operative times are longer, requires higher doses of analgesia, and is associated with a higher incidence of wound infection. **Recommendation 4.3** We recommend conventional three-port laparoscopic appendectomy over single-incision laparoscopic appendectomy, as the conventional laparoscopic approach is associated with shorter operative times, less postoperative pain, and lower incidence of wound infection [QoE: High; Strength of recommendation: Strong; 1A].

#### Q.4.4: Does laparoscopic single-incision surgery confer any advantage over the three-trocar technique in performing laparoscopic appendectomy for pediatric patients with acute appendicitis?

In children, two recent RCTs showed that SILA is feasible with an acceptable margin of safety, although it does not produce any significant difference in terms of analgesic use and length of hospital stay [145], and it is associated with longer operative times and more severe surgical trauma compared with the three-port technique, as measured by CRP and IL-6 levels [146]. In the large meta-analysis by Zhang et al., no significant differences were observed between SILA and CLA with respect to the incidence of total postoperative complications, IAA, ileus, wound hematoma, length of hospital stay, or the frequency of use of additional analgesics. However, SILA was associated with a higher incidence of SSI compared with three-port LA and required a longer operative time [147].

Karam et al. conducted a retrospective study with the aim to compare surgical outcomes of children with AA treated with the transumbilical laparoscopically assisted appendectomy (TULAA) versus the CLA and showed that TULAA had a shorter operative time (median, 40 vs 67 min; *P* < 0.001), a shorter length of stay (median, 20 vs 23 h; *P* < 0.001), and lower costs (median $6266 vs $8927; *P* < 0.001), even if SSI rate was slightly higher in the TULAA group (6% vs 4%; *P* = 0.19) [148].

Sekioka et al. reported that mean operative time was significantly shorter in TULAA than in CLA for both uncomplicated and complicated AA. In addition, complication rates in complicated AA were significantly lower in TULAA than in CLA. Moreover, the postoperative hospital stay was significantly shorter in TULAA than in CLA [149].

**Statement 4.4** In children with acute appendicitis, the single incision/transumbilical extracorporeal laparoscopic-assisted technique is as safe as the laparoscopic three-port technique. **Recommendation 4.4** In pediatric patients with acute appendicitis and favorable anatomy, we suggest performing single-incision/transumbilical extracorporeal laparoscopic assisted appendectomy or traditional three-port laparoscopic appendectomy based on local skills and expertise [QoE: Low; Strength of recommendation: Weak; 2C].

#### Q.4.5: Is outpatient laparoscopic appendectomy safe and feasible for patients with uncomplicated acute appendicitis?

In the USA, outpatient LA protocols are currently applied at multiple institutions with the aim to reduce the length of stay and decrease overall health care costs for AA. Results from these experiences demonstrate that outpatient LA can be performed with a high rate of success, low morbidity, and low readmission rate in the case of non-perforated AA [150]. In the study by Frazee et al., 484 patients with uncomplicated AA were managed as outpatients. Only seven patients (1.2%) were readmitted after outpatient management for transient fever, nausea/vomiting, migraine headache, urinary tract infection, partial small bowel obstruction, and deep venous thrombosis. There were no mortalities or reoperations. Including the readmissions, overall success with outpatient management was 85% [151]. The recent RCT by Trejo-Avila et al. stated that ERAS implementation for appendectomy is associated with a significantly shorter LOS, allowing for the ambulatory management of patients with uncomplicated AA. The authors concluded that ambulatory LA is safe and feasible with similar rates of morbidity and readmissions compared with conventional care [152].

**Statement 4.5** Outpatient laparoscopic appendectomy for uncomplicated acute appendicitis is feasible and safe without any difference in morbidity and readmission rates. It is associated with potential benefits of earlier recovery after surgery and lower hospital and social costs. **Recommendation 4.5** We suggest the adoption of outpatient laparoscopic appendectomy for uncomplicated appendicitis, provided that an ambulatory pathway with well-defined ERAS protocols and patient information/consent are locally established [QoE: Moderate; Strength of recommendation: Weak; 2B].

#### Q.4.6: Is laparoscopic appendectomy indicated over open appendectomy in specific patient groups?

LA is a safe and effective method to treat AA in specific settings such as the elderly and the obese. LA can be recommended for patients with complicated AA even with higher risk categories. In the retrospective cohort study by Werkgartner et al. investigating the benefits of LA in patients with high peri- and postoperative risk factors (ASA 3 and 4), LA was associated with slightly longer operative times and shorter hospital stay. Overall complications, graded according to the Clavien-Dindo classification, were slightly more frequent in patients after LA, whereas severe complications occurred more frequently in patients after OA [153]. For high-risk patients, LA has proven to be safe and feasible and was also associated with decreased rates of mortality, postoperative morbidity, and shorter hospitalization.

In the recent meta-analysis by Wang et al., 12 studies with 126,237 elderly patients in the LA group and 213,201 patients in the OA group were analyzed. Postoperative mortality, as well as postoperative complications and SSI were reduced following LA. IAA rate was similar between LA and OA. Duration of surgery was longer following LA, and the length of hospital stay was shorter following LA [154].

Results from the American College of Surgeons NSQIP (pediatric database) demonstrated that obesity was not found to be an independent risk factor for postoperative complications following LA. Although operative time was increased in obese children, obesity did not increase the likelihood of 30-day postoperative complications [155].

LA also appears to be a safer alternative approach to OA in obese adult patients. In the systematic review by Dasari et al. including seven retrospective cohort studies and one randomized controlled trial, LA in obese patients was associated with reduced mortality (RR 0.19), reduced overall morbidity (RR 0.49), reduced superficial SSI (RR 0.27), and shorter operating times and postoperative length of hospital stay, compared to OA [156].

Despite concerns about the safety of LA during pregnancy being highlighted over the last 10 years due to a possible increase in fetal loss rate, more recent large systematic reviews and meta-analyses of comparative studies concluded that it is not reasonable to state that LA in pregnant women might be associated with a greater risk of fetal loss. Twenty-two comparative cohort studies were included in the pooled analysis by Lee et al., which involved 4694 women of whom 905 underwent LA and 3789 underwent OA. Fetal loss was significantly higher among those who underwent LA compared with those who underwent OA, with a pooled OR of 1.72. However, the sensitivity analysis showed that the effect size was influenced by one of the studies because its removal resulted in there being no significant difference between LA and OA with respect to the risk of fetal loss (OR 1.16). A significant difference was not evident between LA and OA with respect to preterm delivery (OR 0.76), and patients who underwent LA had shorter hospital stays and a lower SSI risk compared with those who underwent OA [157].

**Statement 4.6** Laparoscopic appendectomy seems to show relevant advantages compared to open appendectomy in obese adult patients, older patients, and patients with comorbidities. Laparoscopic appendectomy is associated with reduced mortality, reduced overall morbidity, reduced superficial wound infections, and shorter operating times and postoperative length of hospital stay in such patients. **Recommendation 4.6** We suggest laparoscopic appendectomy in obese patients, older patients, and patients with high peri- and postoperative risk factors [QoE: Moderate; Strength of recommendation: Weak; 2B].

**Statement 4.7** Laparoscopic appendectomy during pregnancy is safe in terms of risk of fetal loss and preterm delivery and it is preferable to open surgery as associated to shorter length of hospital stay and lower incidence of surgical site infection. **Recommendation 4.7** We suggest laparoscopic appendectomy should be preferred to open appendectomy in pregnant patients when surgery is indicated. Laparoscopy is technically safe and feasible during pregnancy where expertise of laparoscopy is available [QoE: Moderate; Strength of recommendation: Weak; 2B].

#### Q.4.7: Does aspiration alone confer clinical advantages over lavage and aspiration for patients with complicated acute appendicitis?

The best available evidence suggests that peritoneal irrigation with normal saline during LA does not provide additional benefits compared with suction alone in terms of IAA, SSI, and length of stay, but it may prolong the operative time.

The recent meta-analysis by Siotos et al., including more than 2500 patients from five studies, has shown that the use of irrigation, despite adding 7 min to the duration of the operation, overall did not demonstrate a significant decrease in IAA. Both for the adult and pediatric subpopulations, the use of irrigation was associated with a non-significant lower odd of IAA [158].

In the same way, the large meta-analysis by Hajibandeh et al. (three RCTs and two retrospective observational studies included) demonstrated that there was no difference between peritoneal irrigation and suction alone in terms of IAA rate, SSI, and length of stay. These results remained consistent when RCTs, adult patients, and pediatric patients were analyzed separately [159]. However, the quality of the best available evidence on this point is moderate; therefore, high-quality, adequately powered randomized studies are required to provide a more robust basis for definite conclusions.

**Statement 4.8** Peritoneal irrigation does not have any advantage over suction alone in complicated appendicitis in both adults and children. The performance of irrigation during laparoscopic appendectomy does not seem to prevent the development of IAA and wound infections in neither adults nor pediatric patients. **Recommendation 4.8** We recommend performing suction alone in complicated appendicitis patients with intra-abdominal collections undergoing laparoscopic appendectomy [QoE: Moderate; Strength of recommendation: Strong; 1B].

#### Q.4.8: Does the type of mesoappendix dissection technique (endoclip, endoloop, electrocoagulation, Harmonic Scalpel, or LigaSure) produce different clinical outcomes for patients with acute appendicitis undergoing appendectomy?

Simplified and cost-effective techniques for LA have been described. They use either two endoloops, securing the blood supply, or a small number of endoclips.

In the case of an inflamed and edematous mesoappendix, it has been suggested that the use of LigaSure^TM^, especially in the presence of gangrenous tissue, may be advantageous [160, 161]. Despite the potential advantages, LigaSure ^TM^ represents a high-cost option and it may be logical using endoclips if the mesoappendix is not edematous. Diamantis et al. compared LigaSure^TM^ and Harmonic Scalpel with monopolar electrocoagulation and bipolar coagulation: the first two caused more minimal thermal injury of the surrounding tissue than other techniques [162]. Recently, significantly higher thermal damage was found on the mesoappendix and appendiceal base in patients treated with LigaSure ^TM^ than in patients for whom Harmonic Scalpel was used during LA [163].

Monopolar electrocoagulation, being safe, quick, and related to very low rates of complications and conversion to OA, can be considered the most cost-effective method for mesoappendix dissection in LA [164]. A recent retrospective cohort study by Wright et al. has proposed that the use of a single stapler line for transection of the mesoappendix and appendix as a safe and efficient technique that results in reduced operative duration with excellent surgical outcomes [165].

**Statement 4.9** There are no clinical differences in outcomes, length of hospital stay, and complication rates between the different techniques described for mesentery dissection (monopolar electrocoagulation, bipolar energy, metal clips, endoloops, LigaSure, Harmonic Scalpel, etc.). **Recommendation 4.9** We suggest the use of monopolar electrocoagulation and bipolar energy as they are the most cost-effective techniques, whereas other energy devices can be used depending on the intra-operative judgment of the surgeon and resources available [QoE: Moderate; Strength of recommendation: Weak; 2B].

#### Q.4.9: Does the type of stump closure technique (stapler or endoloop, ligation or invagination of the stump) produce different clinical outcomes for patients with acute appendicitis undergoing appendectomy?

The stump closure may vary widely in practice and the associated costs can be significant. While earlier studies initially reported advantages with routine use of endostaplers in terms of complication and operative times, more recent studies have repeatedly demonstrated no differences in intra- or postoperative complications between either endostapler or endoloops stump closure [166].

Recent evidence shows that the use of Hem-O-Lok (HOL) clips is safe and reduced the costs of the procedure in comparison to the use of endoloops. In the study by Al-Termini et al., HOL clip use was associated with lower overall complications rate compared with endoloops. The minimum endoloop cost per single appendectomy was $273.13, while HOL clip cost was $32.14 [167].

The multicenter prospective observational study by Van Rossem et al. has demonstrated that the infectious complication rate is not influenced by the type of appendicular stump closure when comparing endoloops or an endostapler. Median operating time was not different between endoloop and endostapler use (42.0 vs 44.0 min) and no significant effect of stump closure type was observed for any infectious complication or IAA. In multivariable analysis, complicated AA was identified as the only independent risk factor for IAA [168].

In the same way, the large systematic review and meta-analysis by Ceresoli et al. showed that in complicated AA, the stump closure technique did not affect outcomes. A total of 5934 patients from 14 studies were included in the analysis. Overall, endostapler use was associated with a similar IAA rate but a lower incidence of SSI, whereas the length of stay and readmission and reoperation rates were similar [169].

The most recent Cochrane review comparing mechanical appendix stump closure (stapler, clips, or electrothermal devices) versus ligation (endoloop, Roeder loop, or intracorporeal knot techniques) for uncomplicated AA included eight RCTs encompassing 850 participants. Five studies compared titanium clips versus ligature, two studies compared an endoscopic stapler device versus ligature, and one study compared an endoscopic stapler device, titanium clips, and ligature. No differences in total complications, intra-operative complications, or postoperative complications between ligature and all types of mechanical devices were found. However, the analyses of secondary outcomes revealed that the use of mechanical devices saved approximately 9 min of the total operating time when compared with the use of a ligature, even though this result did not translate into a clinically or statistically significant reduction in inpatient hospital stay [170].

Recently, 43 randomized controlled trials enrolling over 5,000 patients were analyzed in the network meta-analysis by Antoniou et al. The authors concluded that the use of suture ligation of the appendix in LA seems to be superior to other methods for the composite parameters of organ/space and superficial operative site infection [171].

Current evidence suggests that polymeric clips are an effective and cost-efficient method for stump closure in LA for AA. In the recent meta-analysis by Knight et al. including over 700 patients, polymeric clips were found to be the cheapest method (€20.47 average per patient) and had the lowest rate of complications (2.7%) compared to other commonly used closure methods. Meanwhile, operative time and duration of in-patient stay were similar between groups [172].

Many studies compared the simple ligation and the stump inversion and no significant difference was found. Eleven RCTs (2634 patients) were included in the systematic review and meta-analysis by Qian et al. Postoperative pyrexia and infections were similar between simple ligation and stump inversion groups, respectively, but the former group had a shorter operative time, less incidence of postoperative ileus, and quicker postoperative recovery. The clinical results revealed that simple ligation was significantly superior to stump inversion [173].

**Statement 4.10** There are no clinical advantages in the use of endostaplers over endoloops for stump closure for both adults and children in either simple or complicated appendicitis, except for a lower incidence of wound infection when using endostaplers in children with uncomplicated appendicitis. Polymeric clips may be the cheapest and easiest method (with shorter operative times) for stump closure in uncomplicated appendicitis. **Recommendation 4.10** We recommend the use of endoloops/suture ligation or polymeric clips for stump closure for both adults and children in either uncomplicated or complicated appendicitis, whereas endostaplers may be used when dealing with complicated cases depending on the intra-operative judgment of the surgeon and resources available [QoE: Moderate; Strength of recommendation: Strong; 1B].

**Statement 4.11** Simple ligation should be preferred to stump inversion, either in open or laparoscopic surgery, as the major morbidity and infectious complications are similar. Simple ligation is associated with shorter operative times, less postoperative ileus and quicker recovery. **Recommendation 4.11** We recommend simple ligation over stump inversion either in open and laparoscopic appendectomy [QoE: High; Strength of recommendation: Strong; 1A].

#### Q.4.10: Is the use of abdominal drains recommended after appendectomy for complicated acute appendicitis in adult patients?

The updated 2019 Cochrane review on the issue included six RCTs (521 participants), comparing abdominal drainage and no drainage in patients undergoing emergency OA for complicated AA. The authors found that there was insufficient evidence to determine the effects of abdominal drainage and no drainage on intra-peritoneal abscess or for SSI at 14 days. The increased risk of a 30-day overall complication rate in the drainage group was rated as very low-quality evidence, as well as the evidence that drainage increases hospital stay by 2.17 days compared to the no drainage group. Thus, there is no evidence for any clinical improvement by using abdominal drainage in patients undergoing OA for complicated AA [174].

Low-quality studies have reported that routine drainage has not proven its utility and seems to cause more complications, higher length of hospital stay, and transit recovery time [175]. In the large retrospective cohort study by Schlottmann et al. the placement of intra-abdominal drains in complicated AA did not present benefits in terms of reduced IAA and even lengthened hospital stay [176].

**Statement 4.12** In adult patients, the use of drains after appendectomy for perforated appendicitis and abscess/peritonitis should be discouraged. Drains are of no benefit in preventing intra-abdominal abscess and lead to longer length of hospitalization, and there is also low-quality evidence of increased 30-day morbidity and mortality rates in patients in the drain group. **Recommendation 4.12** We recommend against the use of drains following appendectomy for complicated appendicitis in adult patients [QoE: Moderate; Strength of recommendation: Strong; 1B].

#### Q.4.11: Is the use of abdominal drains recommended after appendectomy for complicated acute appendicitis in pediatric patients?

The prophylactic use of abdominal drainage after LA for perforated AA in children does not prevent postoperative complications and may be associated with negative outcomes.

Aneiros Castro et al. retrospectively analyzed 192 pediatric patients (mean age of 7.77 ± 3.4 years) undergoing early LA for perforated AA and reported that there were no statistically significant differences between the drain and no drain groups in the rate of IAA, SSI, and bowel obstruction. However, drains were statistically associated with an increased requirement for antibiotic and analgesic medication, fasting time, operative time, and length of hospital stay [177].

**Statement 4.13** The prophylactic use of abdominal drainage after laparoscopic appendectomy for perforated appendicitis in children does not prevent postoperative complications and may be associated with negative outcomes. **Recommendation 4.13** We suggest against the prophylactic use of abdominal drainage after laparoscopic appendectomy for complicated appendicitis in children [QoE: Low; Strength of recommendation: Weak; 2C].

#### Q.4.12: What are the best methods to reduce the risk of SSI in open appendectomies with contaminated/dirty wounds?

Wound edge protectors significantly reduce the rate of SSI in open abdominal surgery. The systematic review and meta-analysis by Mihaljevic et al. (16 randomized controlled trials including 3695 patients investigating wound edge protectors published between 1972 and 2014) showed that wound edge protectors significantly reduced the rate of SSI (RR 0.65). A similar effect size was found in the subgroup of patients undergoing colorectal surgery (RR 0.65). Of the two common types of wound protectors, double-ring devices were found to exhibit a greater protective effect (RR 0.29) than single-ring devices (RR 0.71) [178].

The use of ring retractors showed some evidence of SSI reduction (RR 0.44) in the meta-analysis by Ahmed et al., which included four RCTs with 939 patients. On subgroup analysis, ring retractor was more effective in more severe degrees of appendiceal inflammation (contaminated group) [179].

A recent RCT comparing primary and delayed primary wound closure in complicated AA showed that the superficial SSI rate was lower in patients who underwent primary wound closure than delayed primary wound closure (7.3% vs 10%), although the risk difference of − 2.7% was not statistically significant. Postoperative pain, length of stay, recovery times, and quality of life were nonsignificantly different with corresponding risk differences of 0.3, − 0.1, − 0.2, and 0.02, respectively. However, costs for primary wound closure were lower than delayed primary wound closure [180].

In the RCT by Andrade et al. comparing skin closure with a unique absorbable intradermal stitch and traditional closure technique (non-absorbable separated stitches), OA skin closure with the former has shown to be safe, with a reduced seroma and abscess incidence and an equivalent dehiscence and superficial SSI incidence. Furthermore, the relative risk of complications with traditional skin closure was 2.91 higher, compared to this new technique [181].

**Statement 4.14** The use of wound ring protectors shows some evidence of surgical site infection reduction in open appendectomy, especially in case of complicated appendicitis with contaminated/dirty wounds. **Recommendation 4.14** We recommend wound ring protectors in open appendectomy to decrease the risk of SSI [QoE: Moderate; Strength of recommendation: Strong; 1B].

**Statement 4.15** Delayed primary skin closure increases the length of hospital stay and overall costs in open appendectomies with contaminated/dirty wounds and does not reduce the risk of SSI. Subcuticular suture seems preferable in open appendectomy for acute appendicitis as it is associated with a lower risk of complications (surgical site infection/abscess and seroma) and lower costs. **Recommendation 4.15** We recommend primary skin closure with a unique absorbable intradermal suture for open appendectomy wounds [QoE: Moderate; Strength of recommendation: Weak; 2B].

### Topic 5: Intra-operative grading of acute appendicitis

#### Q.5.1: What is the value of scoring systems for intra-operative grading of acute appendicitis?

There is considerable variability in the intra-operative classification of AA. In the multicenter cohort study by Strong et al. involving 3,138 patients, the overall disagreement between the surgeon and the pathologist was reported in 12.5% of cases (moderate reliability, *k* 0.571). Twenty-seven percent of appendices assessed as normal by the surgeon revealed inflammation at histopathological assessment, while 9.6% of macroscopically appearing inflamed AA revealed to be normal [182].

In 2018, a survey among Dutch surgeons demonstrated that a clear standard of care is missing both in patient selection and in determining the length of antibiotic treatment following appendectomy. However, the authors assessed the inter-observer variability in the classification of AA during laparoscopy and demonstrated that agreement was minimal for both the classification of AA (*κ* score 0.398) and the decision to prescribe postoperative antibiotic treatment (*κ* score 0.378) [183].

The definition of complicated AA varies among studies. Apart from the common component of perforation, it may or may not also include non-perforated gangrenous AA, the presence of a fecalith and/or AA in the presence of pus, or purulent peritonitis, or abscess.

Although most surgeons agree that AA with perforation, intra-abdominal abscess, or purulent peritonitis can be defined as complicated AA, for which postoperative antibiotic therapy is indicated, there is still a considerable variation in the indications for prolonged antibiotic therapy after appendectomy, and the antibiotic regimen that should be used [184].

As the intra-operative classification of AA dictates the patient’s postoperative management, such variation in practice may influence clinical outcomes, and standardization may impact the appropriate use of antibiotics worldwide given the issue of rising antimicrobial resistance.

In order to evaluate the appendix during diagnostic laparoscopy, in 2013, Hamminga et al. proposed the LAPP (Laparoscopic APPendicitis) score (six criteria), with a single-center prospective pilot study (134 patients), reporting high positive and negative predictive values (99% and 100%, respectively) [185]. In 2015, Gomes et al. proposed a grading system for AA that incorporates clinical presentation, imaging, and laparoscopic findings. The system, encompassing four grades (0 = normal looking appendix, 1 = inflamed appendix, 2 = necrosis, 3 = inflammatory tumor, 4 = diffuse peritonitis) provides a standardized classification to allow more uniform patient stratification for AA research and to aid in determining optimal management according to the grade of the disease [186].

In 2018, the WSES grading system was validated in a prospective multicenter observational study, performed in 116 worldwide surgical departments from 44 countries over a 6-month period, which showed that 3.8% of patients had grade 0, while 50.4% had grade 1, 16.8% grade 2a, 3.4% grade 2b, 8.8% grade 3a, 4.8% grade 3b, 1.9% grade 3c, and 10.0% grade 4. About half of the patients were grade 1 (inflamed appendix), and this is probably the most common situation for an emergency surgeon [186, 187].

In 2014, the AAST also proposed a system for grading the severity of emergency general surgery diseases based on several criteria encompassing clinical, imaging, endoscopic, operative, and pathologic findings, for eight commonly encountered gastrointestinal conditions, including AA, ranging from grade I (mild) to grade V (severe) [188]. In 2017, Hernandez et al. validated this system in a large cohort of patients with AA, showing that increased AAST grade was associated with open procedures, complications, and length of stay. AAST grade in emergency for AA determined by preoperative imaging strongly correlated with operative findings [189]. In 2018, the same researchers assessed whether the AAST grading system corresponded with AA outcomes in a US pediatric population. Results showed that increased AAST grade was associated with increased Clavien-Dindo severity of complications and length of hospital stay [190].

Moreover, increasing anatomic severity, as defined by AAST grade, has shown to be associated with increasing costs. Length of stay exhibited the strongest association with costs, followed by AAST grade, Clavien-Dindo Index, age-adjusted Charlson score, and surgical wound classification [191]. In 2019, a study by Mällinen et al. corroborated the known clinical association of an appendicolith to complicated AA. The study’s purpose was to assess differences between uncomplicated CT confirmed AA and AA presenting with appendicolith with two prospective patient cohorts. Using multivariable logistic regression models adjusted for age, gender, and symptom duration, statistically significant differences were detected in the depth of inflammation ≤ 2.8 mm (adjusted OR 2.18 (95% CI 1.29–3.71, *P* = 0.004), micro-abscesses (adjusted OR 2.16 (95% CI 1.22–3.83, *P* = 0.008), the number of eosinophils and neutrophils ≥ 150/mm^2^ (adjusted OR 0.97 (95% CI 0.95–0.99, *P* = 0.013), and adjusted OR 3.04 (95% CI 1.82–5.09, *P* < 0.001, respectively) between the two groups of patients [108].

The Sunshine Appendicitis Grading System score (SAGS) can be used to simply and accurately classify the severity of AA, to independently predict the risk of intra-abdominal collection and guide postoperative antibiotic therapy [192].

Based on the results of a large retrospective cohort study, Farach et al. concluded that in children operative findings are more predictive of clinical course than histopathologic results. The authors found there was poor agreement between intra-operative findings and histopathologic findings, and, although 70% of patients with intra-operative findings of uncomplicated AA were labeled as complex pathology, 86% followed a fast track protocol (same-day discharge) with a low complication rate (1.7%) [193].

**Statement 5.1** The incidence of unexpected findings in appendectomy specimens is low. The intra-operative diagnosis alone is insufficient for identifying unexpected disease. From the currently available evidence, routine histopathology is necessary. **Recommendation 5.1** We recommend routine histopathology after appendectomy [QoE: Moderate; Strength of recommendation: Strong; 1B].

**Statement 5.2** Operative findings and intra-operative grading seem to correlate better than histopathology with morbidity, overall outcomes and costs, both in adults and children. Intra-operative grading systems can help the identification of homogeneous groups of patients, determining optimal postoperative management according to the grade of the disease and ultimately improve utilization of resources. **Recommendation 5.2** We suggest the routine adoption of an intra-operative grading system for acute appendicitis (e.g., WSES 2015 grading score or AAST EGS grading score) based on clinical, imaging and operative findings [QoE: Moderate; Strength of recommendation: Weak; 2B].

#### Q.5.2: Should the macroscopically normal appendix be removed during laparoscopy for acute right iliac fossa pain when no other explanatory pathology is found?

Laparoscopic management of normal appendix still represents a dilemma for the surgeon, as no high-level evidence-based recommendations are available to date.

The Society of American Gastrointestinal and Endoscopic Surgeons (SAGES) 2010 guidelines stated that, if no other pathology is identified, the decision to remove the appendix should be considered, but based on the individual clinical scenario [194]. In the same way, the European Association of Endoscopic Surgery (EAES) 2016 guidelines recommended performing an appendectomy in the case of a normal appearing appendix during surgery for suspected AA [195].

Intra-operative macroscopic distinction between a normal appendix and AA during surgery can be challenging. Several studies have shown a 19% to 40% rate of pathologically abnormal appendix in the setting of no visual abnormalities [182, 196]. Therefore, the risk of leaving a potentially abnormal appendix must be weighed against the risk of appendectomy in each individual scenario. Cases of postoperative symptoms requiring reoperation for appendectomy have been described in patients whose normal appendix was left in place at the time of the original procedure. The risks of leaving in situ an apparently normal appendix are related to later AA, subclinical or endo-appendicitis with persisting symptoms, and missed appendiceal malignancy.

According to the retrospective study by Grimes et al., including 203 appendectomies performed with normal histology, fecaliths may be the cause of right iliac fossa pain in the absence of obvious appendiceal inflammation. In this study, the policy of routine removal of a normal-looking appendix at laparoscopy in the absence of any other obvious pathology appeared to be an effective treatment for recurrent symptoms [197]. In the same way, Tartaglia et al. supported an appendectomy in patients undergoing laparoscopy for acute right lower quadrant abdominal pain even when the appendix appears normal on visual inspection, based on the results of a study in which 90% of the removed normal-looking appendices at laparoscopy for abdominal pain and no other intra-abdominal acute disease harbored inflammatory changes at the definitive pathology [198].

Recently, Sørensen et al. performed a retrospective cohort analysis of patients who underwent a diagnostic laparoscopy due to clinical suspicion of AA where no other pathology was found, and the appendix was not removed. Of the 271 patients included, 56 (20.7%) were readmitted with right iliac fossa pain after a median time of 10 months. Twenty-two patients (8.1%) underwent a new laparoscopic procedure, and the appendix was removed in 18 patients, of which only one showed histological signs of inflammation. Based on results from this study, the authors did not consider that it is necessary to remove a macroscopic normal appendix during laparoscopy for clinically suspected AA [199]. This year, Allaway et al. published the results of a single-centre retrospective case note review of patients undergoing LA for suspected AA. Patients were divided into positive and negative appendectomy groups based on histology results. The authors reported an overall negative appendectomy rate of 36.0% among 1413 patients who met inclusion criteria (904 in the positive group and 509 in the negative group). Morbidity rates (6.3% vs 6.9%; *P* = 0.48) and types of morbidity were the same for negative appendicectomy and uncomplicated AA, and there was no significant difference in complication severity or length of stay (2.3 vs 2.6 days; *P* = 0.06) between negative appendicectomy and uncomplicated AA groups [200].

The 2014 Cochrane review on the use of laparoscopy for the management of acute lower abdominal pain in women of childbearing age showed that laparoscopy was associated with an increased rate of specific diagnoses. A significant difference favoring the laparoscopic procedure in the rate of removal of normal appendix compared to open appendectomy was found [201].

**Statement 5.3** Surgeon's macroscopic judgment of early grades of acute appendicitis is inaccurate and highly variable. The variability in the intra-operative classification of appendicitis influences the decision to prescribe postoperative antibiotics and should be therefore prevented/avoided. **Recommendation 5.3** We suggest appendix removal if the appendix appears “normal” during surgery and no other disease is found in symptomatic patients [QoE: Low; Strength of recommendation: Weak; 2C].

### Topic 6: Management of perforated appendicitis with phlegmon or abscess

#### Q.6.1: Is early appendectomy an appropriate treatment compared with delayed appendectomy for patients with perforated acute appendicitis with phlegmon or abscess?

The optimal approach to complicated AA with phlegmon or abscess is a matter of debate.

In the past, immediate surgery has been associated with a higher morbidity if compared with conservative treatment, while the non-surgical treatment of appendicular abscess or phlegmon has been reported to succeed in over 90% of patients, with an overall risk of recurrence of 7.4% and only 19.7% of cases of abscess requiring percutaneous drainage [202].

The meta-analysis by Similis et al. (including 16 non-randomized retrospective studies and one non-randomized prospective study for a total of 1572 patients, of whom 847 treated with conservative treatment and 725 with appendectomy) revealed that conservative treatment was associated with significantly less overall complications (wound infections, abdominal/pelvic abscesses, ileus/bowel obstructions, and re-operations) if compared to immediate appendectomy [203].

In the large series from the National Inpatient Sample (NIS) by Horn et al., 25.4% of a total of 2,209 adult patients with appendiceal abscesses who received drains failed conservative management and underwent operative intervention [204].

Current evidence shows that surgical treatment of patients presenting with appendiceal phlegmon or abscess is preferable to NOM with antibiotic oriented treatment in the reduction of the length of hospital stay and need for readmissions when laparoscopic expertise is available [205]. In the retrospective study by Young et al., early appendectomy has shown superior outcomes compared with initial NOM. Of 95 patients presenting with complicated AA, 60 underwent early appendectomy, and 35 initially underwent NOM. All patients who experienced failed NOM (25.7%) had an open operation with most requiring bowel resection. Early appendectomy demonstrated a lower incidence of bowel resection (3.3% vs 17.1%, *P* = 0.048) when compared to all patients initially undergoing NOM [206].

Recently, the cumulative meta-analysis by Gavriilidis et al. has shown a more widespread use of the laparoscopic approach for the management of complicated AA. Although overall complications, abdominal/pelvic abscesses, wound infections, and unplanned procedures were significantly lower in the conservative treatment cohort in the general analysis, on the contrary, the subgroup analysis of three RCTs revealed no significant difference in abdominal/pelvic abscesses (OR 0.46). High-quality RCTs demonstrated shorter hospital stay by 1 day for the LA cohort compared to conservative treatment [207].

According to the results of the Cochrane review published by Cheng et al. in 2017, it is unclear whether early appendectomy shows any benefit in terms of complications compared to delayed appendectomy for people with appendiceal phlegmon or abscess. The review included only two RCTs with a total of 80 participants. The comparison between early versus delayed open appendectomy for appendiceal phlegmon included 40 participants (pediatric and adults), randomized either to early appendectomy (appendectomy as soon as appendiceal mass resolved within the same admission, *n* = 20) or to delayed appendectomy (initial conservative treatment followed by interval appendectomy 6 weeks later, *n* = 20). There was insufficient evidence to determine the effect of using either early or delayed open appendectomy on overall morbidity (RR 13.00), the proportion of participants who developed wound infection (RR 9.00), or fecal fistula (RR 3.00). Even the quality of evidence for increased length of hospital stay and time away from normal activities in the early appendectomy group was of very low quality. The comparison between early versus delayed laparoscopic appendectomy for appendiceal abscess included 40 pediatric patients, randomized either to early appendectomy (emergent laparoscopic appendicectomy, *n* = 20) or to delayed appendectomy (initial conservative treatment followed by interval laparoscopic appendicectomy 10 weeks later, *n* = 20). Health-related quality of life score measured at 12 weeks after appendectomy was higher in the early appendectomy group than in the delayed appendectomy group, but the quality of evidence was very low [208].

The high-quality RCT by Mentula et al. (not included in the Cochrane review), conversely, demonstrated that LA in experienced hands is a safe and feasible first-line treatment for appendiceal abscess. In this study, early LA was associated with fewer readmissions and fewer additional interventions than conservative treatment, with a comparable hospital stay. Patients in the laparoscopy group had a 10% risk of bowel resection and 13% risk of incomplete appendectomy. There were significantly fewer patients with unplanned readmissions following LA (3% versus 27%, *P* = 0.026). Additional interventions were required in 7% of patients in the laparoscopy group (percutaneous drainage) and 30% of patients in the conservative group (appendectomy). Conversion to open surgery was required in 10% of patients in the laparoscopy group and 13% of patients in the conservative group. The rate of uneventful recovery was 90% in the laparoscopy group versus 50% in the conservative group (*P* = 0.002) [209].

Luo et al. analyzed the outcomes of 1,225 patients under 18 years of age who had non-surgical treatment for an appendiceal abscess between 2007 and 2012 in Taiwan. The authors compared outcomes of percutaneous drainage with antibiotics or antibiotics alone. Of 6,190 children having an appendiceal abscess, 1,225 patients received non-operative treatment. Patients treated with percutaneous drainage and antibiotics had a significantly lower rate of recurrent AA, significantly smaller chance of receiving an interval appendectomy, and significantly fewer postoperative complications after the interval appendectomy than those without percutaneous drainage treatment. In addition, patients treated with percutaneous drainage were significantly less indicated to receive an interval appendectomy later [210].

Two recent meta-analyses addressed the role of early appendectomy in children with appendiceal phlegmon or abscess. The meta-analysis by Fugazzola et al. found that children with appendiceal abscess/phlegmon reported better results in terms of complication rate and readmission rate if treated with NOM [211]. Similarly, the meta-analysis by Vaos et al. reported that NOM was associated with lower rates of complications and wound infections, whereas the development of IAA and postoperative ileus was not affected by the treatment of choice [212]. In both the meta-analyses, early appendectomy was associated with reduced length of hospital stay.

**Statement 6.1** Non-operative management is a reasonable first-line treatment for appendicitis with phlegmon or abscess. Percutaneous drainage as an adjunct to antibiotics, if accessible, could be beneficial, although there is a lack of evidence for its use on a routine basis. Laparoscopic surgery in experienced hands is a safe and feasible first-line treatment for appendiceal abscess, being associated with fewer readmissions and fewer additional interventions than conservative treatment, with a comparable hospital stay. **Recommendation 6.1** We suggest non-operative management with antibiotics and—if available—percutaneous drainage for complicated appendicitis with a periappendicular abscess, in settings where laparoscopic expertise is not available [QoE: Moderate; Strength of recommendation: Weak; 2B].

**Statement 6.2** Operative management of acute appendicitis with phlegmon or abscess is a safe alternative to non-operative management in experienced hands and may be associated with shorter LOS, reduced need for readmissions, and fewer additional interventions than conservative treatment. **Recommendation 6.2** We suggest the laparoscopic approach as treatment of choice for patients with complicated appendicitis with phlegmon or abscess where advanced laparoscopic expertise is available, with a low threshold for conversion. [QoE: Moderate; Strength of recommendation: Weak; 2B].

#### Q.6.2: Is interval appendectomy always indicated for patients with acute appendicitis following successful NOM?

The reported rate of recurrence after non-surgical treatment for perforated AA and phlegmon is up to 12% [213]. In order to avoid this quite high chance of recurrence, some authors recommend routine elective interval appendectomy following initial conservative management. However, this procedure is associated with a non-negligible rate of morbidity of 12.4% [202]. The systematic review by Hall et al., including three retrospective studies for a total of 127 cases of non-surgical treatment of appendix mass in children, showed that after successful non-operative treatment the risk of recurrent AA was found to be 20.5%. Overall, the complications reported included SSI, prolonged postoperative ileus, hematoma formation, and small bowel obstruction, but the incidence of any individual complication was not determined [23].

In the recent systematic review by Darwazeh et al., interval appendectomy and repeated NOM in the case of recurrence of appendiceal phlegmon were associated with similar morbidity. However, elective interval appendectomy was related to additional operative costs to prevent recurrence in only one of eight patients, such as not to justify the routine performance of appendectomy [213].

In the same way, Rushing et al., who found a risk of recurrence of 24.3% in patients, managed with NOM for appendiceal abscess or phlegmon and recommended against routine interval appendectomy in otherwise asymptomatic patients [214]. The CHINA RCT recently compared the outcomes of active observation versus interval appendectomy after successful NOM of an appendix mass in children. Results showed that more than three-quarters of children could avoid appendectomy during early follow-up after successful NOM of an appendix mass. The proportion of children with histologically proven recurrent AA under active observation was 12%, and the proportion of children with severe complications related to interval appendicectomy was 6%.

Although the risk of complications after interval appendectomy was low, adoption of a wait-and-see approach, reserving appendectomy for patients who develop AA recurrence or recurrent symptoms, should be considered a most cost-effective management strategy compared with routine interval appendectomy [215].

In the study by Renteria et al., unexpected malignancy was 3% in the elderly (mean age 66 years) and 1.5% in the young (mean age 39 years) cohorts of patients who underwent appendectomy as primary treatment for AA [216]. Adult patients with complicated AA treated with interval appendectomy can be diagnosed with appendiceal neoplasm in up to 11% of cases, in contrast to 1.5% of the patients who have early appendectomy [217]. Recently, the RCT by Mällinen et al. comparing interval appendectomy and follow-up with MRI after initial successful nonoperative treatment of periappendicular abscess was prematurely terminated owing to ethical concerns following the unexpected finding at the interim analysis of a high rate of neoplasm (17%), with all neoplasms in patients older than 40 years [218]. If this significant rate of neoplasms after periappendicular abscess is validated by future studies, it would argue for routine interval appendectomy in this setting.

**Statement 6.3** The reported rate of recurrence after non-surgical treatment for perforated AA and phlegmon ranges from 12% to 24%. Interval appendectomy and repeated NOM in case of recurrence of appendiceal phlegmon are associated with similar morbidity. However, elective interval appendectomy is related to additional operative costs to prevent recurrence in only one of eight patients, such as not to justify the routine performance of appendectomy. **Recommendation 6.3** We recommend against routine interval appendectomy after NOM for complicated appendicitis in young adults (< 40 years old) and children. Interval appendectomy is recommended for those patients with recurrent symptoms [QoE: Moderate; Strength of recommendation: Strong; 1B].

**Statement 6.4** The incidence of appendicular neoplasms is high (3–17%) in adult patients ≥ 40 years old) with complicated appendicitis. **Recommendation 6.4** We suggest both colonic screening with colonscopy and interval full-dose contrast-enhanced CT scan for patients with appendicitis treated non-operatively if ≥ 40 years old [QoE: Low; Strength of recommendation: Weak; 2C].

### Topic 7: Perioperative antibiotic therapy

#### Q.7.1: Is preoperative antibiotic therapy recommended for patients with acute appendicitis?

In 2001, a Cochrane meta-analysis supported that broad-spectrum antibiotics given preoperatively are effective in decreasing SSI and abscesses. RCTs and non-randomized comparative studies in which any antibiotic regime was compared to placebo in patients undergoing appendectomy were analyzed. Forty-four studies including 9,298 patients were included in this review. Antibiotics were superior to placebo for preventing wound infection and intra-abdominal abscess, with no apparent difference in the nature of the removed appendix [219]. The same final results have been obtained by the 2005 updated version of the review, including 45 studies with 9,576 patients [220]. The timing of pre-operative antibiotics does not affect the frequency of SSI after appendectomy for AA. Therefore, the optimal timing of preoperative antibiotic administration may be from 0 to 60 min before the surgical skin incision [221].

**Statement 7.1** A single dose of broad-spectrum antibiotics given preoperatively (from 0 to 60 min before the surgical skin incision) has been shown to be effective in decreasing wound infection and postoperative intra-abdominal abscess, with no apparent difference in the nature of the removed appendix. **Recommendation 7.1** We recommend a single preoperative dose of broad-spectrum antibiotics in patients with acute appendicitis undergoing appendectomy. We recommend against postoperative antibiotics for patients with uncomplicated appendicitis [QoE: High; Strength of recommendation: Strong; 1A].

#### Q.7.2: Are postoperative antibiotics always indicated in adult patients following appendectomy?

Prospective trials demonstrated that patients with perforated AA should receive postoperative antibiotic treatment, especially if complete source control has not been achieved. Cho et al. recently demonstrated in a large cohort of patients that the role of antibiotic treatment for preventing post-appendectomy IAA seems to be related with achieving intraperitoneal infectious source control. The authors found that the mean durations of postoperative antibiotic therapy were 3.1 days for the non-IAA group and 3.3 days for the IAA group, with no significant difference between the groups [222].

In the large observational study by McGillen et al., patients with complicated AA were significantly more likely to be started on antibiotics after surgery (83.9% versus 33.3%; *P* < 0.001) compared with patients with simple AA. The development of a SSI was significantly associated with a clinical diagnosis of diabetes, the presence of free fluid, abscess, or perforation on pre-operative imaging [223].

The optimal course of antibiotics remains to be identified, but current evidence suggests that longer postoperative courses do not prevent SSI compared with 2 days of antibiotics.

The meta-analysis by Van den Boom et al., including nine studies with more than 2,000 patients with complicated AA, revealed a statistically significant difference in IAA incidence between the antibiotic treatment of ≤ 5 vs > 5 days (OR 0.36), but not between ≤ 3 vs > 3 days (OR 0.81) [224].

A total of 80 patients were enrolled in a recent RCT comparing the outcomes of short (24 h) and the extended (> 24 h) postoperative antibiotic therapy in complicated AA. The overall rate of complications was 17.9% and 29.3% in the short and extended group, respectively (*P* = 0.23). Mean complication index did not differ between the study groups (*P* = 0.29), whereas hospital length of stay was significantly reduced in the short therapy group (61 ± 34 h vs 81 ± 40 h, *P* = 0.005). Based on the results of this RCT, 24 h of antibiotic therapy following appendectomy does not result in worse primary outcomes in complicated AA, but results in a significant reduction in length of hospitalization, with a major cost-saving and antibacterial stewardship benefits [225].

Although discontinuation of antimicrobial treatment should be based on clinical and laboratory criteria, a period of 3–5 days for adult patients is generally sufficient following appendectomy for complicated AA. The 2015 “STOP-IT” RCT by Sawyer et al. on 518 patients with complicated intra-abdominal infection, including also complicated AA, undergoing adequate source control demonstrated that outcomes after fixed-duration antibiotic therapy (approximately 4 days) were similar to those after a longer course of antibiotics (approximately 8 days) that extended until after the resolution of physiological abnormalities [226].

**Statement 7.2** In patients with complicated acute appendicitis, postoperative broad-spectrum antibiotics are suggested, especially if complete source control has not been achieved. For adult patients deemed to require them, discontinuation of antibiotics after 24 h seems safe and is associated with shorter length of hospital stay and lower costs. In patients with intra-abdominal infections who had undergone an adequate source control, the outcomes after fixed-duration antibiotic therapy (approximately 3–5 days) are similar to those after a longer course of antibiotics. **Recommendation 7.2** We recommend against prolonging antibiotics longer than 3–5 days postoperatively in case of complicated appendicitis with adequate source control [QoE: High; Strength of recommendation: Strong; 1A].

#### Q.7.3: Are postoperative antibiotics always indicated in pediatric patients following appendectomy?

A retrospective review conducted by Litz et al. demonstrated that antibiotic administration within 1 h of appendectomy in pediatric patients with AA who receive antibiotics at diagnosis did not change the incidence of postoperative infectious complications [227].

Children with non-perforated AA should receive a single broad-spectrum antibiotic. Second- or third-generation cephalosporins, such as cefoxitin or cefotetan, may be used in uncomplicated cases.

In complicated AA, intravenous antibiotics that are effective against enteric gram-negative organisms and anaerobes including *E. coli* and *Bacteroides* spp. should be initiated as soon as the diagnosis is established. Broader-spectrum coverage is obtained with piperacillin-tazobactam, ampicillin-sulbactam, ticarcillin-clavulanate, or imipenem-cilastatin. For perforated AA, the most common combination is ampicillin, clindamycin (or metronidazole), and gentamicin. Alternatives include ceftriaxone-metronidazole or ticarcillin-clavulanate plus gentamicin, in accordance with the epidemiology of bacteria [228]. Metronidazole is not indicated when broad-spectrum antibiotics such as aminopenicillins with β-lactam inhibitors or carbapenems and select cephalosporins are used [229]. In a recent retrospective cohort study of 24,984 children aged 3 to 18 years, Kronman et al. compared the effectiveness of extended-spectrum versus narrower-spectrum antibiotics for children with AA. The exposure of interest was receipt of systemic extended-spectrum antibiotics (piperacillin ± tazobactam, ticarcillin ± clavulanate, ceftazidime, cefepime, or a carbapenem) on the day of appendectomy or the day after. The primary outcome was 30-day readmission for SSI or repeat abdominal surgery. The authors reported that extended-spectrum antibiotics seem to offer no advantage over narrower-spectrum agents for children with surgically managed acute uncomplicated or complicated AA [230].

Broad-spectrum, single, or double agent therapy is equally efficacious as but more cost-effective than triple agent therapy. It was reported that dual therapy consisting of ceftriaxone and metronidazole only offers a more efficient and cost-effective antibiotic management compared with triple therapy, but prospective studies are required to determine whether this policy is associated with higher rates of wound infections and change in antibiotic therapy [231].

Postoperative antibiotics can be administered orally if the patient is otherwise well enough to be discharged. Arnold et al. conducted a RCT of 82 pediatric patients to compare the effect of home intravenous versus oral antibiotic therapy on complication rates and resource utilization following appendectomy for perforated AA. Fosrty-four patients (54%) were randomized to the IV group and 38 (46%) to the oral group. The study showed no difference in length of stay (4.4 ± 1.5 versus 4.4 ± 2.0 days), postoperative abscess rate (11.6% vs 8.1%), or readmission rate (14.0% vs 16.2%), whereas hospital and outpatient charges were higher in the IV group [232].

Other retrospective cohort studies have confirmed that after apspendectomy for perforated AA in children, oral antibiotics show equivalent outcomes compared with intravenous antibiotics, but with shorter length of hospitalizations and less medical encounters required [233].

Compared to pediatric patients who receive intravenous antibiotics, those who are treated with oral antibiotics have statistically lower rates of repeated US imaging (49.6% vs 35.1%) and PICC placement (98.3% vs 9.1%), whereas the rates of IAA are similar (20.9% vs 16.0%). Moreover, early transition to oral antibiotics allows shorter hospital times and decreased hospital charges, with similar total antibiotic days and readmission rate [234].

**Statement 7.3** Administering postoperative antibiotics orally in children with complicated appendicitis for periods shorter than 7 days postoperatively seems to be safe and it is not associated with increased risk of complications. Early transition to oral antibiotics is safe, effective, and cost-efficient in the treatment of complicated appendicitis in the child. **Recommendation 7.3** We recommend early switch (after 48 h) to oral administration of postoperative antibiotics in children with complicated appendicitis, with an overall length of therapy shorter than seven days [QoE: Moderate; Strength of recommendation: Strong; 1B].

**Statement 7.4** Postoperative antibiotics after appendectomy for uncomplicated acute appendicitis in children seems to have no role in reducing the rate of surgical site infection. **Recommendation 7.4** In pediatric patients operated for uncomplicated acute appendicitis, we suggest against using postoperative antibiotic therapy [QoE: Low; Strength of recommendation: Weak; 2C].

## Conclusions

**Fig. 1:**
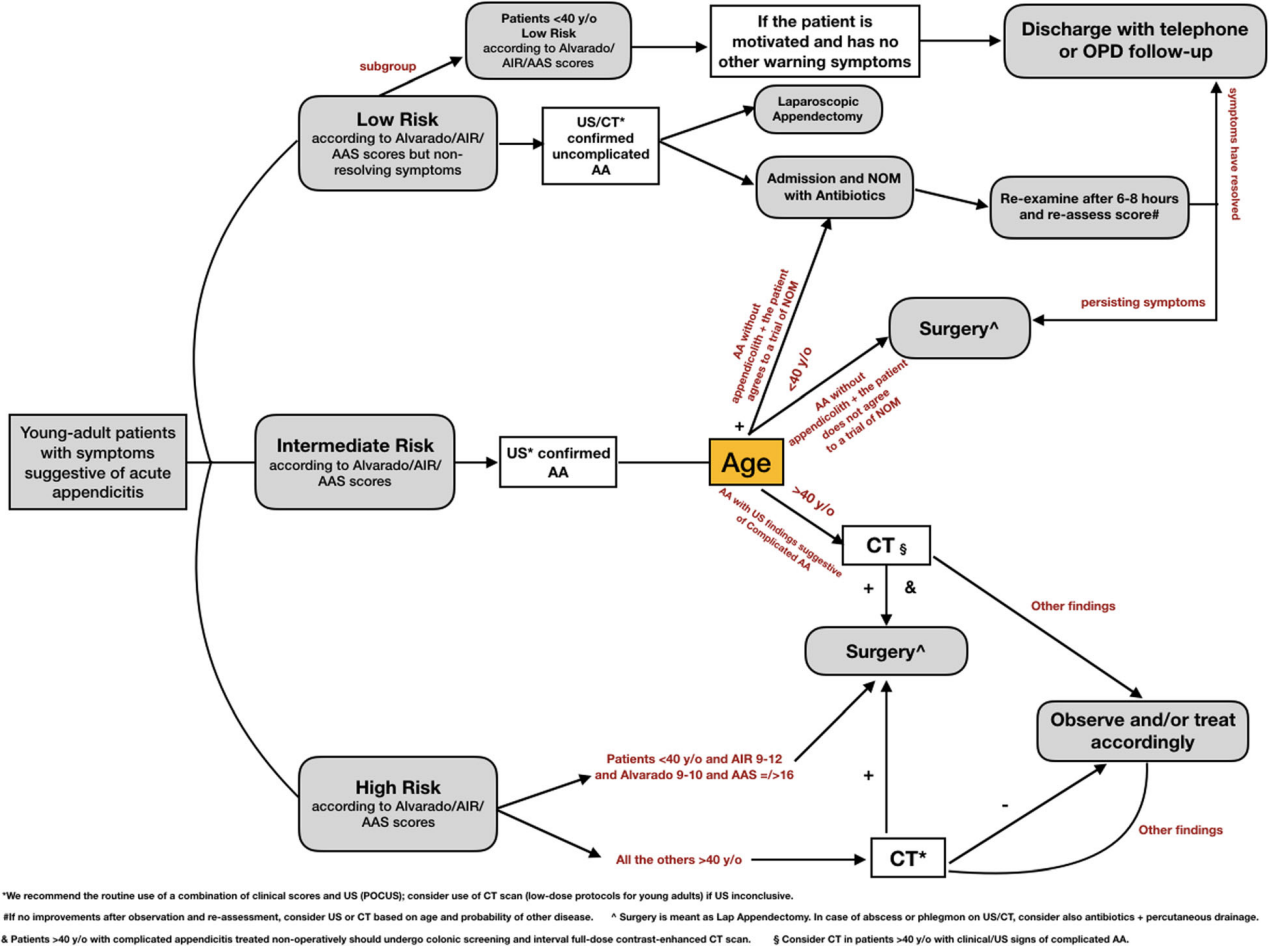
Practical WSES algorithm for diagnosis and treatment of adult patients with suspected acute appendicitis

**Fig. 2:**
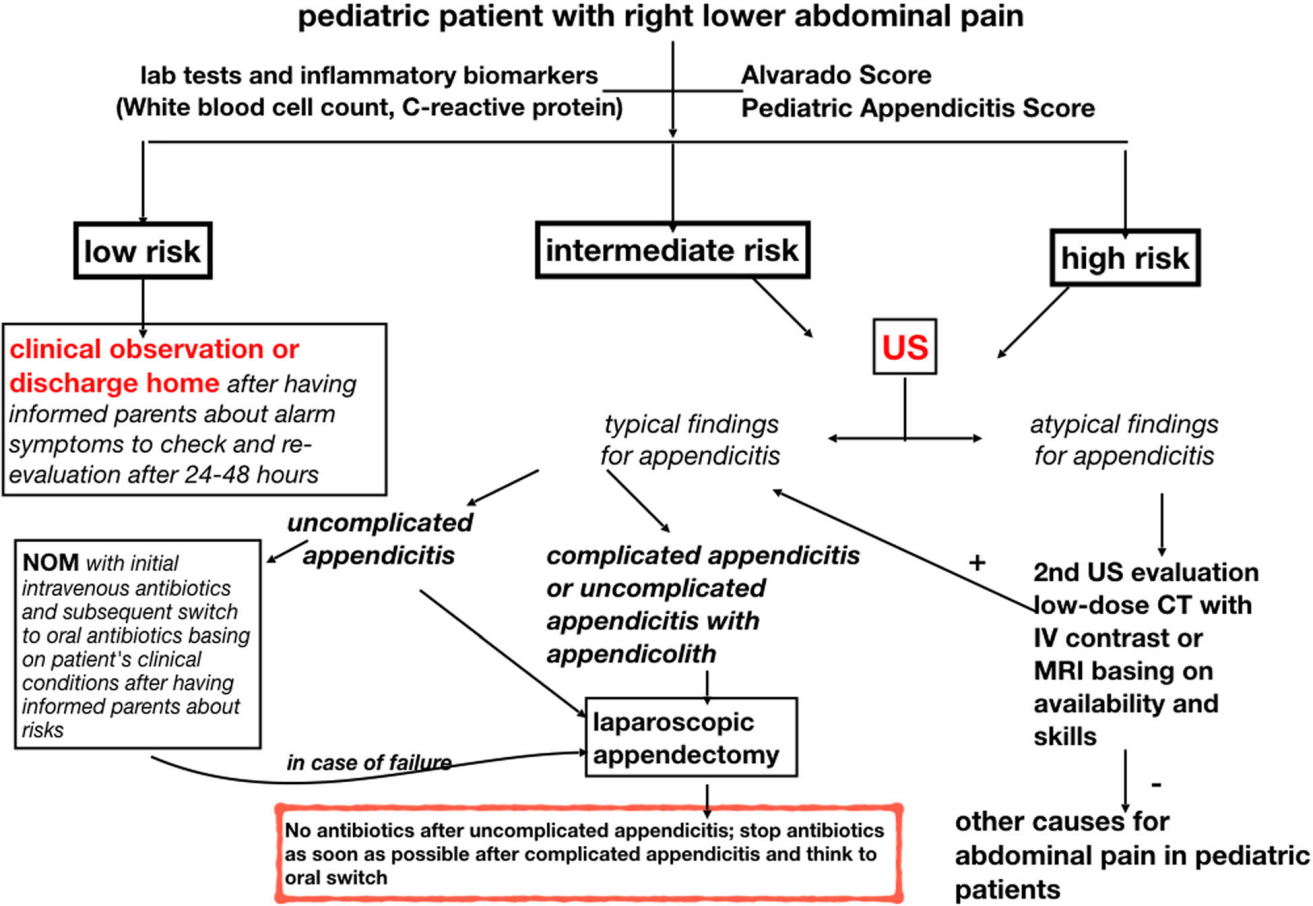
Practical WSES algorithm for diagnosis and treatment of pediatric patients with suspected acute appendicitis

The current evidence-based guidelines are the updated 2020 International Comprehensive Clinical Guidelines for the diagnosis and management of acute appendicitis. After reaching consensus on each of the above mentioned, the panel experts and the scientific committee members developed two WSES flow-chart algorithm for the diagnosis and management of acute appendicits to be used for adults and pediatric patient population, reported respectively in Figs. 1 and 2.

## Supplementary information

**Additional file 1.** Search Syntaxes.

**Additional file 2.**

**Additional file 3.**

**Additional file 4.**

**Additional file 5.**

**Additional file 6.**

## Data Availability

There are no individual author data that reach the criteria for availability.
