# Supplementary material for: Diagnosis and treatment of acute appendicitis: 2020 update of the WSES Jerusalem guidelines
Source: World J Emerg Surg. 2020 Apr 15;15:27. doi: 10.1186/s13017-020-00306-3 (PMC7386163; doi:10.1186/s13017-020-00306-3)
Supplement: Supplementary file 1 — Additional file 1. Search Syntaxes. [file 13017_2020_306_MOESM1_ESM.doc]

**Appendix 1.** Search syntaxes.

| **Topic** | **Search strategies** |
| --- | --- |
| ***Diagnosis*** | 2015:2019[PDAT] AND appendicitis [TI] AND (score [TI] OR scoring system*[TI] OR scoring method*[TI]) |
|  | 2015:2019[PDAT] AND appendicitis [TI] AND (clinical tool [TI] OR indicator[TI] OR predictor[TI] OR model[TI] OR algorithm[TI] OR rule[TI] OR criteria[TI] OR indicator[TI] OR validation OR (score[TI] OR scoring system*[TI] OR scoring method*[TI])) |
|  | 2015:2019[PDAT] AND Appendicitis [TI] AND Imaging [TI]; Appendicitis [TI] AND Ultrasonography [TI]; Appendicitis [TI] AND Ultrasound scan; Appendicitis [TI] AND Computed Tomography [TI]; (Appendicitis) AND (magnetic resonance OR MRI); (Appendicitis)[TI] AND Biochemical markers |
| ***NOM of uncomplicated appendicitis*** | 2015:2019[PDAT] AND ((((antibiotic therapy [MeSH Terms]) OR conservative treatment) OR non operative treatment) AND appendectomy) OR appendicectomy) AND appendicitis |
| ***Timing of appendectomy and in-hospital delay*** | 2015/04:2019/04[PDAT] AND (time*[TI] OR early[TI] OR delayed[TI]) AND (appendicitis[TI] OR appendectom*[TI]) |
|  | 2015/04:2019/04[PDAT] AND (time-to-treatment[TI] OR early[TI] OR delayed[TI]) AND (appendicitis[TI] OR appendectom*[TI]) |
| ***Surgical treatment*** | Appendicitis/surgery"[Mesh] AND 2015/04[PDAT] : 2019/04[PDAT] AND (Review[ptyp] OR systematic[sb] OR Consensus Development Conference[ptyp] OR Meta-Analysis[ptyp] OR Randomized Controlled Trial[ptyp] OR Observational Study[ptyp]) |
|  | appendicitis[TI] AND (drain*[TI] OR lavage[TI] OR aspiration[TI] OR suction[TI] OR mesoappendix[TI]) AND 2015/04:2019/04[PDAT] |
|  | ((appendicitis[TI] AND (surgery[TI] OR "surgical management"[TI] OR "surgical treatment"[TI])) OR appendectomy[TI]) AND (elderly[TI] OR obese[TI] OR child*[TI] OR pregnant[TI]) AND 2015/04:2019/04[PDAT] |
| ***Intra-operative grading of acute appendicitis*** | 2015/04:2019/04[PDAT] AND intra-operative[TI] OR "scoring system*"[TI] OR score*[TI] OR grad*[TI] OR "scoring method*[TI]) AND appendicitis[TI] |
|  | (((((("2015/04"[Date - Publication] : "2019/04"[Date - Publication])) AND "intraoperative"[Title/Abstract]) AND score*[Title]) OR grade*[Title]) OR grading system*[Title]) AND appendicitis[Title] |
| ***Management of perforated appendicitis with phlegmon or abscess*** | (("Appendicitis/complications"[Mesh]) OR "complicated appendicitis"[TIAB]) AND ("Conservative Treatment"[Mesh] OR "non operative management"[TIAB]) |
|  | 2015/04:2019/04[PDAT] AND "complicated appendicitis"[TI] AND (non-operative management[Title] OR conservative management[Title] OR non-operative treatment[Title] OR conservative treatment[Title]) |
|  | 2015/04:2019/04[PDAT] AND appendicitis[TI] AND (non-operative management[Title] OR conservative management[Title] OR non-operative treatment[Title] OR conservative treatment[Title]) |
| ***Antibiotic prophylaxis and postoperative antibiotic treatment*** | ("Appendicitis/therapy"[Mesh]) AND ("Antibiotic Prophylaxis"[Mesh] OR antibiotic*) AND (time[TIAB] OR duration[TIAB]) AND 2015/04:2019/04[PDAT] |
|  | ("Appendicitis/therapy"[Mesh]) AND ("Antibiotic Prophylaxis"[Mesh] OR antibiotic*[TI]) AND (appendectomy[TI] OR surgery[TI]) AND 2015/04:2019/04[PDAT] |
|  | ("Antibiotic Prophylaxis"[Mesh] OR antibiotic*[TI]) AND (after[TI] OR before[TI]) AND (appendectomy[TI] OR "appendicitis/surgery"[MeSH] OR appendicitis[TI]) AND 2015/04:2019/04[PDAT] |
|  | ("microbial prophylaxis"[TI] OR antibiotic*[TI] OR antibiotic prophylaxis[MeSH]) AND (preoperative[TI] OR postoperative[TI]) AND (appendectomy[TI] OR "appendicitis/surgery"[MeSH] OR appendicitis[TI]) AND 2015/04:2019/04[PDAT] |
