# Supplementary figures and images for: Diagnosis and treatment of acute appendicitis: 2020 update of the WSES Jerusalem guidelines
Source: World J Emerg Surg. 2020 Apr 15;15:27. doi: 10.1186/s13017-020-00306-3 (PMC7386163; doi:10.1186/s13017-020-00306-3)

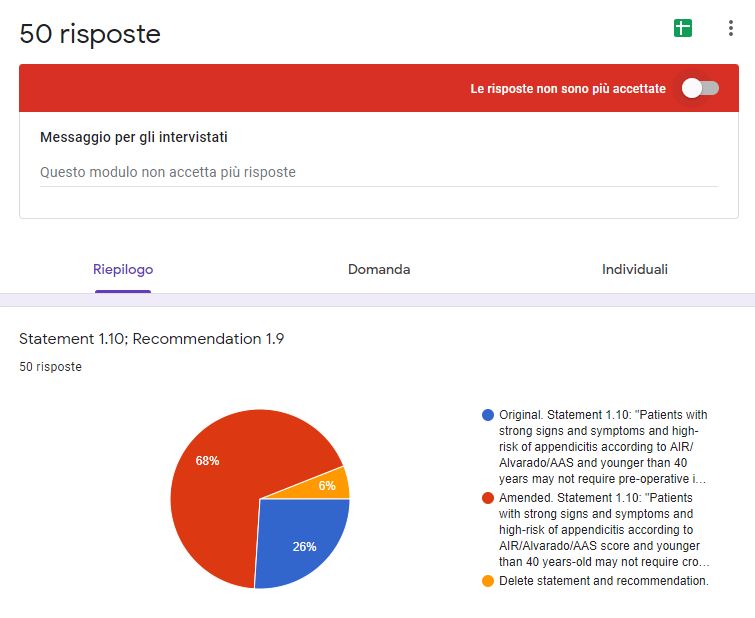

Supplement: Supplementary file 2 — Additional file 2. [file 13017_2020_306_MOESM2_ESM.jpg]

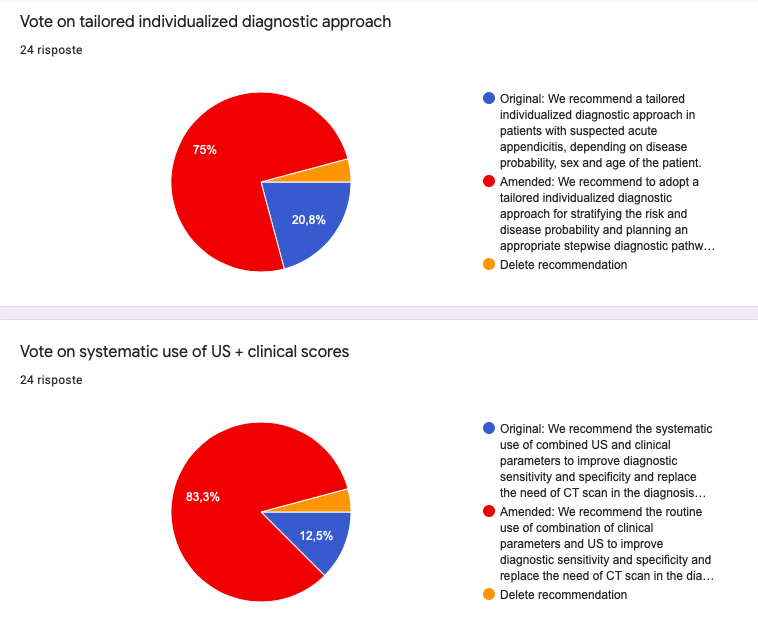

Supplement: Supplementary file 3 — Additional file 3. [file 13017_2020_306_MOESM3_ESM.png]

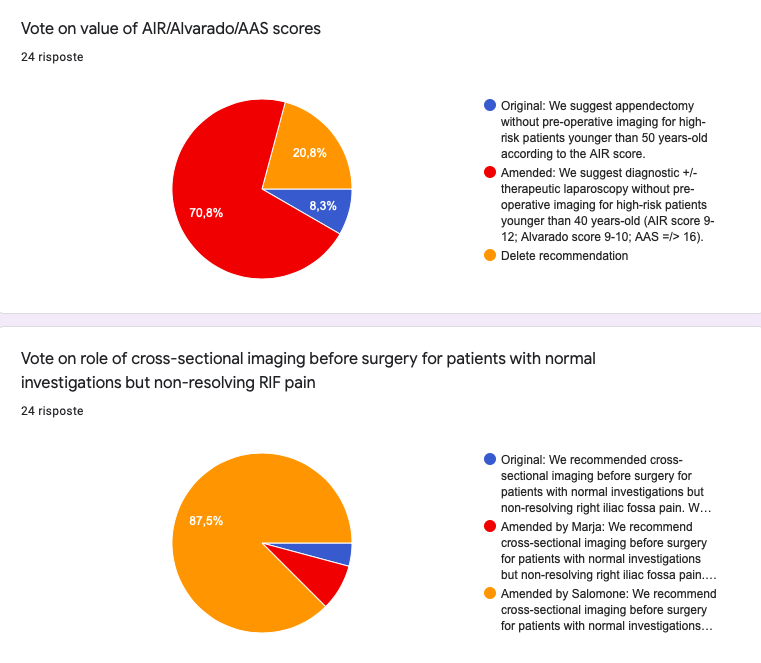

Supplement: Supplementary file 4 — Additional file 4. [file 13017_2020_306_MOESM4_ESM.png]

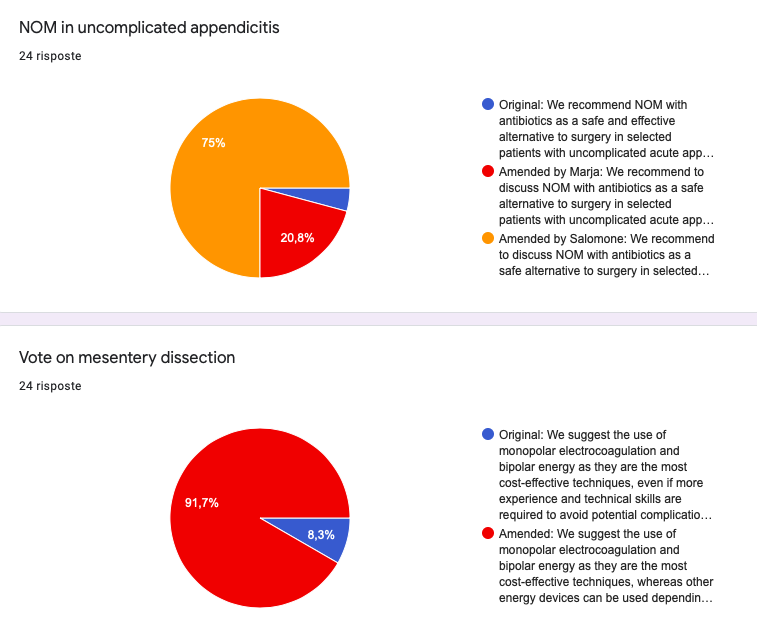

Supplement: Supplementary file 5 — Additional file 5. [file 13017_2020_306_MOESM5_ESM.png]

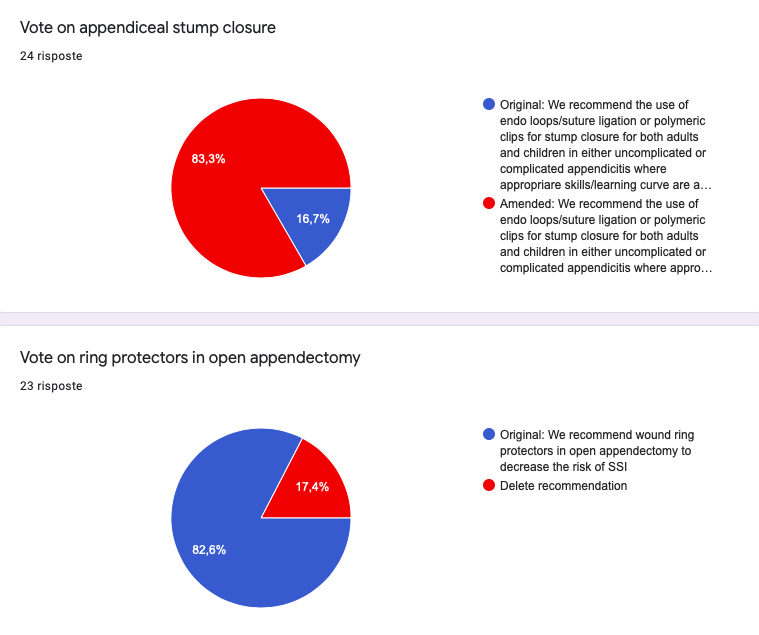

Supplement: Supplementary file 6 — Additional file 6. [file 13017_2020_306_MOESM6_ESM.png]
